# Dancing with the Tides: Fluctuations of Coastal Phytoplankton Orchestrated by Different Oscillatory Modes of the Tidal Cycle

**DOI:** 10.1371/journal.pone.0049319

**Published:** 2012-11-14

**Authors:** Anouk N. Blauw, Elisa Benincà, Remi W. P. M. Laane, Naomi Greenwood, Jef Huisman

**Affiliations:** 1 Aquatic Microbiology, Institute for Biodiversity and Ecosystem Dynamics, University of Amsterdam, Amsterdam, The Netherlands; 2 Marine and Coastal Systems, Deltares, Delft, The Netherlands; 3 Marine Observations Systems, Centre for Environment, Fisheries and Aquaculture Science (CEFAS), Lowestoft, Suffolk, United Kingdom; University of Otago, New Zealand

## Abstract

Population fluctuations are often driven by an interplay between intrinsic population processes and extrinsic environmental forcing. To investigate this interplay, we analyzed fluctuations in coastal phytoplankton concentration in relation to the tidal cycle. Time series of chlorophyll fluorescence, suspended particulate matter (SPM), salinity and temperature were obtained from an automated measuring platform in the southern North Sea, covering 9 years of data at a resolution of 12 to 30 minutes. Wavelet analysis showed that chlorophyll fluctuations were dominated by periodicities of 6 hours 12 min, 12 hours 25 min, 24 hours and 15 days, which correspond to the typical periodicities of tidal current speeds, the semidiurnal tidal cycle, the day-night cycle, and the spring-neap tidal cycle, respectively. During most of the year, chlorophyll and SPM fluctuated in phase with tidal current speed, indicative of alternating periods of sinking and vertical mixing of algal cells and SPM driven by the tidal cycle. Spring blooms slowly built up over several spring-neap tidal cycles, and subsequently expanded in late spring when a strong decline of the SPM concentration during neap tide enabled a temporary “escape” of the chlorophyll concentration from the tidal mixing regime. Our results demonstrate that the tidal cycle is a major determinant of phytoplankton fluctuations at several different time scales. These findings imply that high-resolution monitoring programs are essential to capture the natural variability of phytoplankton in coastal waters.

## Introduction

What drives fluctuations in population abundances? In the 1920s the famous zoologist Charles Elton argued that population fluctuations of many birds and mammals are most likely due to climatic fluctuations [1]. Shortly thereafter, however, mathematical models of Lotka [2] and Volterra [3] and laboratory experiments by Gause [4] demonstrated that species interactions can also generate population fluctuations, even in the absence of external forcing. Since that time, one of the key challenges for ecologists has been to disentangle the complex interplay between intrinsic population dynamics and environmentally-driven variation [5]–[7].

This interplay between intrinsic population processes and external forcing is exemplified by the plankton of freshwater and marine ecosystems. Theory and experiments have shown that plankton communities can display striking fluctuations, and even chaos, under constant conditions without external forcing [8]–[12]. Such non-equilibrium dynamics can limit the predictability of plankton abundances. For instance, Benincà et al. [12] estimated that the predictability of species fluctuations in an experimental plankton community was limited to a time horizon of only 15–30 days. In addition, plankton communities are also very sensitive to variation in environmental conditions [13]–[17]. Therefore, a major question is how environmental forcing interacts with the intrinsic population fluctuations in plankton communities.

Environmental forcing by the tidal cycle is an important driver of phytoplankton variability in coastal waters [18]–[21]. The tidal cycle is characterized by periodic fluctuations at several time scales. Systems with a semidiurnal tide, like the North Sea, show horizontal displacement of water masses with a periodicity of 12 hours and 25 min. This horizontal motion generates maxima in tidal current speeds and turbulent mixing with a periodicity of 6 hours and 12 min. Other important tidal components include the spring-neap tidal cycle with a periodicity of 15 days and the apogee-perigee cycle with a periodicity of 28 days. The latter cycle is caused by the moon’s elliptic orbit, which enhances the tidal range during perigee (when the moon is closest to Earth) and reduces it during apogee (when the moon is farthest from Earth) [22]. In addition to the tidal cycle, coastal phytoplankton will also be exposed to other environmental variation in, e.g., solar irradiance, temperature and wind mixing.

We hypothesize that these different sources of phytoplankton variability can be distinguished by investigating the time scales of phytoplankton fluctuations. For instance, phytoplankton fluctuations with a periodicity of 6 hours 12 min indicate an alternation between entrainment of sinking phytoplankton into the surface layer during high tidal current speeds and settlement of sinking phytoplankton during tidal slacks. A periodicity of 12 hours 25 min indicates changes in phytoplankton concentration due to horizontal transport of different water masses. A 24 hour periodicity would signal environmental forcing by the day-night cycle (e.g., diurnal stratification), which would be very different from the 24 hour 50 min periodicity of a mixed semidiurnal tide. At longer time scales, the spring-neap cycle and apogee-perigee cycle generate variation in the intensity of tidal mixing that could affect phytoplankton populations.

Furthermore, we hypothesize that the impact of environmental forcing on phytoplankton population dynamics will depend on the relative magnitude of intrinsic population growth versus environmental forcing. For instance, settling and resuspension of phytoplankton in shallow coastal waters depends on the vertical mixing intensity generated by wind action and tidal motion [23], [24]. Hence, in case of strong tidal forcing in comparison to the phytoplankton growth rate, we hypothesize that phytoplankton concentrations will fluctuate in phase with the intensity of tidal mixing. Conversely, in case of weak tidal forcing, the phytoplankton dynamics may be largely governed by intrinsic population growth. Because light availability is often a limiting factor in turbid coastal waters [25], reduced turbidity during calm conditions at neap tide may provide suitable light conditions for phytoplankton growth. Hence, in this case, phytoplankton concentrations are more likely to fluctuate in anti-phase with the intensity of tidal mixing.

To investigate these hypotheses, we analyze phytoplankton fluctuations in a high-resolution time series obtained from an automated mooring station in the coastal North Sea. The mooring measured chlorophyll fluorescence, suspended particulate matter (SPM), nitrate, salinity, temperature and irradiance at a high temporal resolution for a period of nine years. Earlier analysis of the first year of this time series showed that the phytoplankton spring bloom was initiated by an improved light availability in spring, due to a combination of enhanced solar radiation and reduced SPM concentrations [26]. This indicates that settlement of suspended particles may play an important role at this station. However, this earlier analysis focused on the seasonal dynamics, but did not investigate phytoplankton and SPM variability in relation to the tidal regime. We apply wavelet analysis [27]–[29], which is an advanced statistical technique ideally suited to quantify the time scales of the phytoplankton fluctuations and their coherence with SPM and the tidal cycle. Our results reveal how fluctuations of coastal phytoplankton are driven by different oscillatory components of the tidal cycle.

## Methods

### Automated Measurements

**Figure 1:**
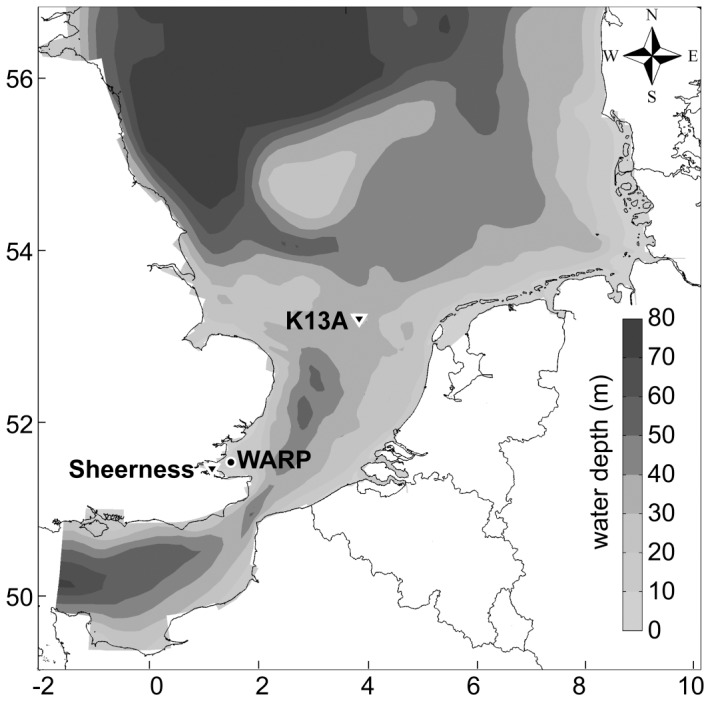
Map of the southern North Sea with the monitoring stations. Phytoplankton, SPM, nutrient, light, salinity and temperature were measured by a SmartBuoy (circle) at mooring station Warp. Tidal data were obtained from tide gauges (triangles) at station Sheerness and station K13A.

We analyzed data of mooring station Warp, located in the coastal waters of the southern North Sea near the Thames Estuary (Fig. 1). The main characteristics of this mooring station are summarized in Table 1. The site has a water depth of 15 m, and an average tidal range of 4.3 m. Vertical profiles of water density measured at this station are generally uniform over depth, indicative of well-mixed waters without thermohaline stratification (Fig. S1).

At the mooring station an automated measuring platform, called a ‘SmartBuoy’, has been deployed from 30 November 2000 onwards, as part of the United Kingdom’s eutrophication monitoring program [30]. The SmartBuoy measured chlorophyll fluorescence, optical backscatter, salinity, temperature and photosynthetically active radiation (PAR) at 1 m depth, using sampling intervals ranging from 12 to 30 min. The nitrate concentration was measured at sampling intervals ranging from 1 to 24 hours. We have analyzed SmartBuoy data from the years 2001–2009. The data can be downloaded from the website http://www.cefas.defra.gov.uk. More information on the methodology and application of SmartBuoys can be found in Kröger et al. [31], Greenwood et al. [32] and Nechad et al. [33]. Seasonal phytoplankton bloom dynamics and biogeochemistry at this station in 2001 have been described by Weston et al. [26].

Chlorophyll fluorescence was measured with a Seapoint fluorometer (Seapoint Inc.). Fluorescence data were calibrated against chlorophyll concentrations in water samples taken during monthly service visits to the mooring stations. Chlorophyll concentrations were measured by filtering known sample volumes through glass fiber filters (GF/F; Whatman) in triplicate. Pigments were immediately extracted in 90% buffered acetone and refrigerated prior to analysis. A Turner Designs Model 10AU filter fluorometer was used to measure the fluorescence of extracted chlorophyll and phaeopigment before and after acidification as described by Tett [34]. The filter fluorometer was calibrated using a solution of pure chlorophyll-a (Sigma-Aldrich) with known concentrations determined spectrophotometrically. Calibration curves of *in situ* Seapoint fluorometer measurements versus extracted chlorophyll concentrations in the water samples had a R^2^ of 0.86 when averaged over all monthly samples. Although chlorophyll fluorescence is a very convenient measurement technique, it is known that the fluorescence signal can be quenched when cells are exposed to high light [19], [35]. This may cause a reduction in chlorophyll fluorescence during the daytime, especially in clear waters at sunny days.

**Table 1:** Main characteristics of the mooring station Warp.

Variable	Value
Latitude – Longitude	51.31 N - 1.02 E
Monitoring years	2001– present
Water depth (m)	15
Tidal range (m)*	4.3
Salinity (-)	33.7 (w) –34.3 (s)
SPM (mg L^−1^)	33.6 (w) –17.3 (s)
Temperature (°C)	6.1 (w) –18.6 (s)

Winter-averaged values (w) of salinity, SPM and temperature are based on mooring data of January – February; summer-averaged values (s) on July – August.

* measured at the nearby station Sheerness.

Optical backscatter was measured with a Seapoint turbidity meter (Seapoint Inc.), and converted to SPM concentration using calibration against monthly water samples. SPM concentrations in the samples were measured by filtering known sample volumes through pre-weighed 0.4 µm polycarbonate filters and subsequent rinsing with 2×50 mL ultrapure water. Filters were then dried in a desiccator at room temperature and weighed until filter weight remained constant. Calibration curves of *in situ* Seapoint turbidity measurements versus SPM concentrations in the water samples had a R^2^ of 0.72 when averaged over all monthly samples.

The concentration of total oxidisable nitrogen (hereafter referred to as nitrate) was measured with a NAS-3X nutrient analyzer (EnviroTech). Salinity and temperature were measured using an FSI CT sensor (Falmouth Scientific Inc.). Salinity measurements were calibrated using a Guildline 8400B salinometer, which had been standardized with IAPSO standard seawater. Downwelling PAR was measured at 1 and 2 m depth using two LiCor (LI-192) underwater quantum sensors (LiCor Biosciences).

The SmartBuoy measurements went through a Quality Assurance protocol within the SmartBuoy Data Management System, checking all data manually for possible sensor malfunction and biofouling. For instance, fluorescence measurements directly before each monthly service were compared with those directly after monthly service to identify signatures of biofouling. Data that looked suspect were flagged and not used in the analyses.

Phytoplankton species composition at the mooring station was determined on an approximately monthly basis. Water samples (150 mL) taken at 1 m depth were preserved with 2.5 mL acidified Lugol’s solution, and analyzed by inverted microscope after 12 hours of settling in a 25-mL glass chamber [36].

Tidal data were obtained from a tide gauge at the nearby coastal station Sheerness (Fig. 1). Inspection of co-tidal lines of the amphidromic system of the North Sea indicated that the tidal wave arrives almost simultaneously at both station Warp and station Sheerness. The tide gauge measured sea water levels at 10 min intervals. The tidal range was calculated as the difference between the maximum and minimum water level of each day. When tidal data from station Sheerness were missing, we resorted to tidal data measured at platform K13A in the central North Sea to approximate the spring-neap cycle at station Sheerness.

### Data Preprocessing

Because the sampling interval of the automated measurements varied between instruments and between years, we calculated hourly averages to obtain a more uniform dataset. We also calculated daily averages of all variables, where the chlorophyll data were confined to measurements made in the dark (i.e., when observed PAR at 1 m depth was <1 *µ*mol quanta m^−2^ s^−1^), to remove possible effects of non-photochemical quenching of chlorophyll fluorescence. We analyzed the hourly averaged and daily averaged time series separately to investigate variability at different time scales.

To analyze periodicities in the time series, we preprocessed the hourly averaged and daily averaged data of chlorophyll concentration, SPM concentration, salinity and temperature following a three-step procedure. First, we log-transformed the data using the natural logarithm. Log transformation reduces the skewness of the data sets, and suppresses the impact of isolated large peaks. Second, we smoothed the log-transformed data using a 3-point moving average filter (i.e., 3-hour and 3-day moving averages for the hourly and daily time series, respectively) to reduce the effect of small-scale variability caused by e.g. measurement noise, intermittent turbulence or local patchiness. We used these small intervals for the moving average filter to avoid smoothing out relevant periodicities of the tidal cycle. Third, we calculated the rates of change from the difference between the data values at time t and time t-Δt, where Δt represents a single time step of one hour or one day. Because we had log-transformed the data, this procedure effectively resulted in a time series of the relative rates of change (‘relative growth rates’) of the measured variables, since.
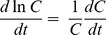



We analyzed the rates of change of the data instead of their concentrations, because concentrations are the result of growth and decay processes during the preceding period whereas instantaneous rates of changes better reflect the conditions at the time of measurement.

### Wavelet Analysis

We used wavelet analysis [27], [28] to identify dominant periodicities in the time series. Wavelet analysis makes use of local periodic functions, known as wavelets. By decomposing the fluctuations of time series into a series of local wavelets (expressed as local wavelet power spectra), one can analyze both the frequency (periodicity) and the timing of the fluctuations. The global wavelet spectrum is the average of all these local wavelet power spectra and is comparable to the power spectrum of traditional spectral analysis. Different wavelet shapes (mother wavelets) are available for wavelet analysis. We have used the Morlet wavelet, which provides a good balance between time and frequency localization [29].

We applied wavelet coherence, an extension of wavelet analysis, to investigate the ‘coherence’ of two time series [28], [29]. Wavelet coherence enables detection of similar periodicities in the fluctuations of two time series and estimates their phase differences.

The statistical significance of periodicities revealed by wavelet analysis was tested by comparing the wavelet power spectrum of our time series against the 95% confidence level of wavelet power spectra generated by red noise, using an autoregressive AR1 model with the same autocorrelation coefficient as our time series [27], [29]. Likewise, we tested whether peaks in the wavelet coherence spectra of two time series were significantly different from the wavelet coherence spectra generated by two red-noise processes with the same autocorrelation coefficients as the two time series. The Matlab scripts for wavelet analysis and wavelet coherence analysis, including significance tests, were developed by Torrence and Compo [27] and Grinsted et al. [29] (available at http://www.pol.ac.uk/home/research/waveletcoherence/).

Our time series contained several data gaps. Small gaps of at most one data point were filled by linear interpolation. Larger gaps could not be filled by interpolation without affecting the results. Therefore, we calculated separate wavelet power spectra for all subsets of the time series with a sufficient number of consecutive data points. Wavelet analysis can accurately detect periodicities up to ∼25% of the length of a time series [28]. Subsets of time series were therefore included in the analysis only if they contained >100 consecutive hours for the hourly averaged data, and >60 consecutive days for the daily averaged data. Global wavelet power spectra of time series were obtained by averaging the local wavelet power spectra at each individual time point over all time points included in the analysis [27].

## Results

### The Data

**Figure 2:**
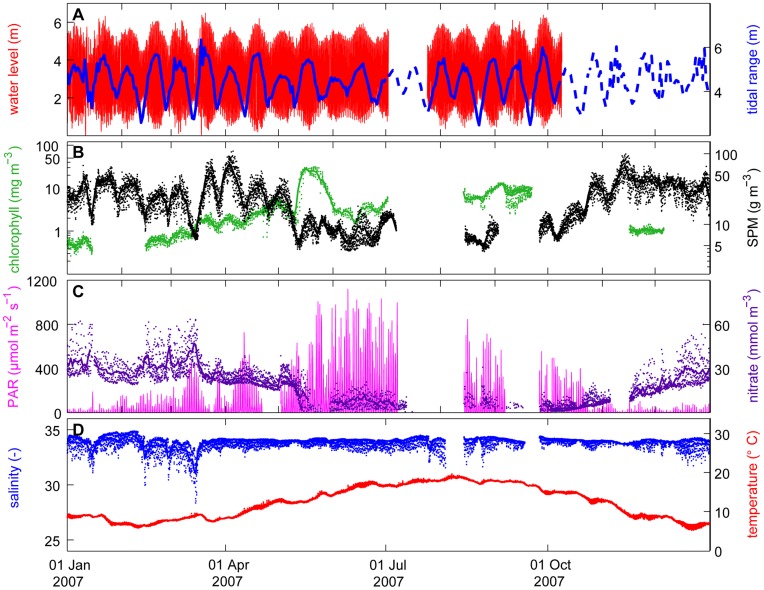
Time series measured in 2007. (A) Water level (red line) and tidal range (blue solid line) at station Sheerness. When tidal data at station Sheerness were missing, we show the tidal range at station K13A (blue dashed line) rescaled to match the tidal range at Sheerness. (B) Chlorophyll concentration (green) and SPM concentration (black). (C) Nitrate concentration (dark purple) and light intensity at 1 m depth (pink). (D) Salinity (blue) and water temperature (red). In (B-D), dots show the hourly averages and lines the daily averages.

Time series of water level, chlorophyll, SPM, nitrate, light intensity, temperature and salinity all displayed strong variability at several different time scales (Fig. 2; Figs. S2, S3, S4, S5, S6, S7, S8, S9). Water level fluctuated at the 12 hour 25 min periodicity of the semidiurnal tidal cycle and the 15-day periodicity of the spring-neap cycle (Fig. 2A). The spring-neap cycle is further illustrated by the time series of the tidal range (difference between high and low tide; blue line in Fig. 2A). SPM concentrations showed considerable within-day variability as well as biweekly fluctuations associated with the spring-neap tidal cycle (Fig. 2B). At the seasonal time scale, SPM concentrations reached higher values in winter than in the summer months. Likewise, the chlorophyll concentration also displayed daily and biweekly fluctuations. The chlorophyll concentration gradually built up over several spring-neap tidal cycles in March-April, resulting in a spring bloom when the SPM concentration strongly declined in mid May (Fig. 2B). The phytoplankton species composition was determined once per month, and consisted of a wide variety of diatoms, dinoflagellates and small flagellates (including *Phaeocystis*). Most abundant were chain-forming diatoms (e.g. *Chaeotoceros*, *Skeletonema*, *Paralia*, *Guinardia* and *Eucampia*), and benthic diatoms (e.g. *Cylindrotheca*, *Plagiogrammopsis* and *Navicula*).

Light intensity (PAR) at 1 m depth followed the expected seasonal pattern, but with distinct daily and biweekly variation (Fig. 2C). High light intensities coincided with low SPM concentrations at neap tide. The light attenuation coefficient (Kd), calculated from PAR measurements at 1 m and 2 m depth, fluctuated in phase with the SPM concentration (Fig. S10). Hence, the SPM concentration can be used as proxy of the turbidity of the water column. The nitrate concentration was highest in winter and early spring, was depleted during the spring bloom in mid May, and remained relatively low during summer (Fig. 2C). Salinity displayed considerable interannual variability (Figs. S2, S3, S4, S5, S6, S7, S8, S9). The timing of the reductions in salinity coincided with neap tides and often with enhanced nitrate concentrations (Fig. 2C,D), indicative of an enhanced influence of nutrient-rich freshwater from the river Thames. Temperature showed a clear seasonal pattern, superimposed by short-term temperature variability (Fig. 2D). The time series all showed several gaps. During these periods observations were lacking or unreliable, due to technical problems or fouling of the sensors.

### The Tidal Cycle

**Figure 3:**
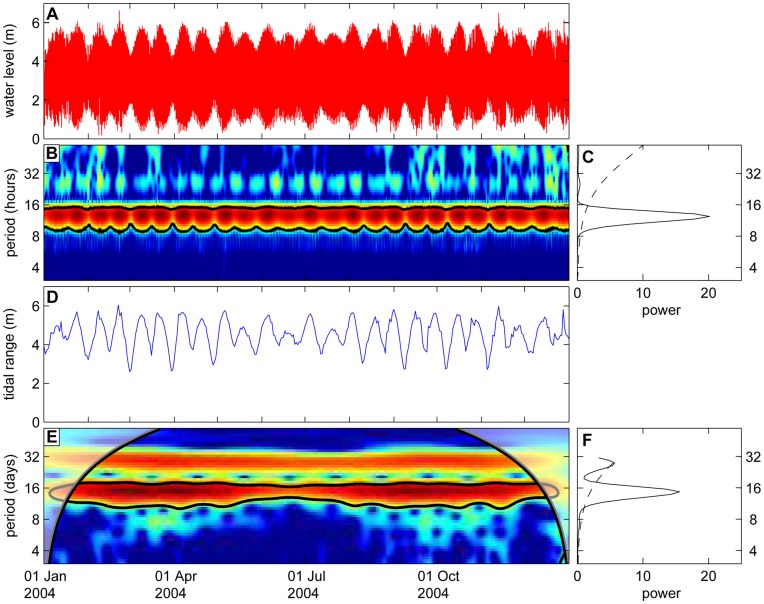
Wavelet analysis of the tidal data. (A) Time series of water level measured at station Sheerness in 2004, with the corresponding (B) wavelet power spectrum and (C) global wavelet power spectrum. (D) Time series of tidal range, with the corresponding (E) wavelet power spectrum and (F) global wavelet power spectrum. The wavelet power spectra in (B) and (E) are presented as contour plots, where the y-axis plots the periodicities in the time series, and the x-axis plots how these periodicities change over time. Color coding indicates the wavelet power, ranging from low power in blue to high power in red. Red areas surrounded by black contour lines enclose significant regions in the wavelet power spectra (i.e., regions of >95% confidence that the local wavelet power exceeds red noise). Shaded areas on the left-hand and right-hand side of the two thick black lines in (E) represent the cone of influence, where edge effects might distort the signal. Results in the cone of influence are therefore excluded from further analysis. Panels (C) and (F) show global wavelet power spectra of the time series (solid lines). Peaks exceeding the 95% confidence level of the corresponding red noise spectra (dashed lines) are significant.

We first analyzed the tidal data measured at station Sheerness (Fig. 3). In line with expectation, wavelet analysis reveals a significant 12-hour periodicity in water level throughout the year (Fig. 3B). This is further illustrated by the global wavelet power spectrum, which has a pronounced peak at a 12-hour periodicity (Fig 3C). The tip of the peak is located at a periodicity of 12 hours 25 min. The peak exceeds the 95% confidence level of red noise (dashed line, Fig. 3C), confirming that the semidiurnal tidal cycle of the North Sea is, indeed, highly significant.

The spring-neap cycle is clearly visible in the time series of the tidal range (Fig. 3D). Close inspection of this time series reveals additional variation in the spring-neap cycle. The tidal range alternates between strong and weak spring tides in May – July, and between strong and weak neap tides in March – April and August – October (Fig. 3D). This pattern is caused by the apogee-perigee cycle. Wavelet analysis of the tidal range confirms the significance of the 15-day periodicity of the spring-neap cycle, while the 28-day periodicity of the apogee-perigee cycle is at the edge of significance (Fig. 3E,F).

### Analysis of Hourly-averaged Time Series

**Figure 4:**
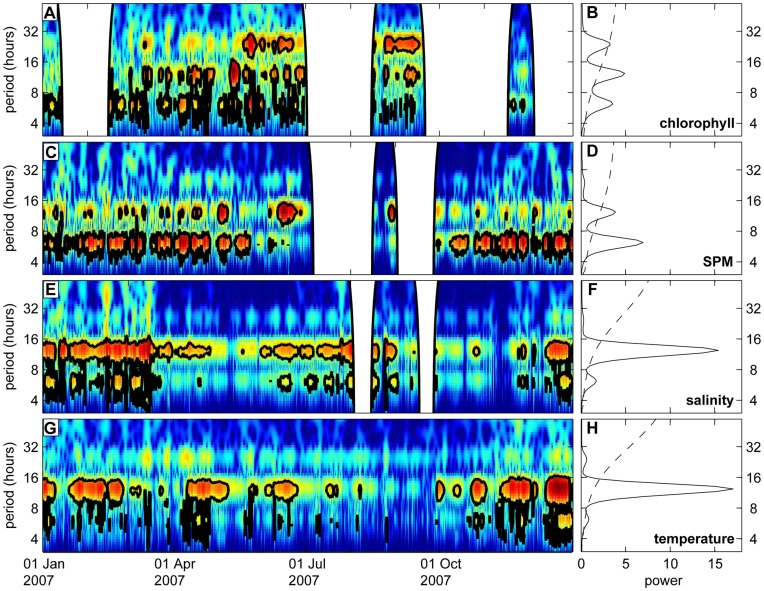
Wavelet analysis of chlorophyll, SPM, salinity and temperature on an hourly time scale. Wavelet power spectra (left panels) and global wavelet spectra (right panels) using hourly averaged data of (A, B) chlorophyll concentration, (C, D) SPM concentration, (E, F) salinity, and (G, H) temperature. The wavelet power spectra show only the year 2007; the global wavelet spectra are based on the complete time series (2001–2009). See the legend of Fig. 3 for an explanation of wavelet power spectra.

Wavelet analysis of the hourly-averaged chlorophyll concentration of the year 2007 reveals significant periodicities of 6 hours 12 min and 12 hours 25 min during most of the year, and an additional 24-hour periodicity in late spring and summer only (Fig. 4A). Likewise, SPM also fluctuated at significant periodicities of 6 hours 12 min and of 12 hours 25 min (Fig. 4C), with highest power at 6-hour periodicity. This is consistent with the 6-hour periodicity in tidal current speeds and, hence, tidal mixing. Conversely, salinity and water temperature showed predominantly 12-hour periodicity (Figs. 4E, G), in agreement with the 12-hour periodicity in the horizontal displacement of water by the tides. There was no significant 24-hour periodicity in water temperature, which indicates that temperature fluctuations due to diel heating and cooling were small compared to fluctuations due to horizontal transport. Analysis of the entire dataset of 2001–2009, summarized in the global wavelet spectra (Figs. 4B, D, F, H), shows the same dominant periodicities as the results for 2007.

**Figure 5:**
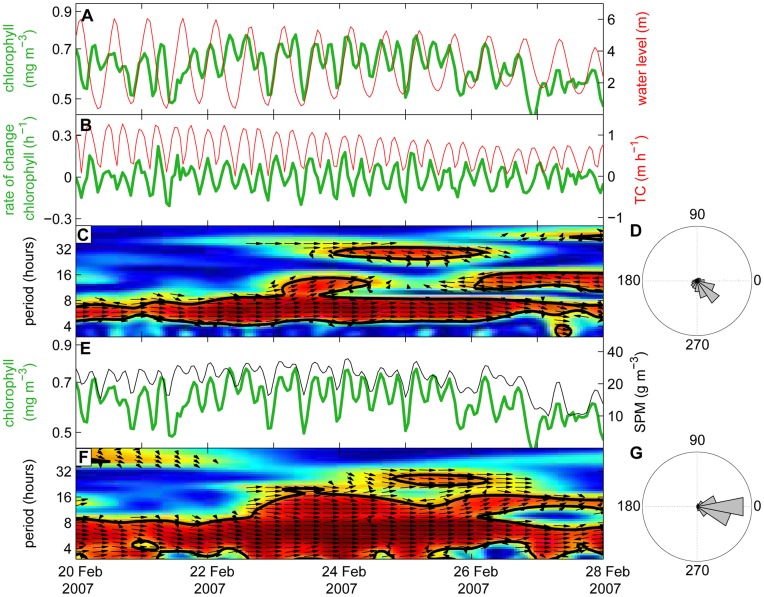
Coherence between fluctuations in chlorophyll, tidal current speed and SPM on an hourly time scale. (A) Time series of hourly averaged chlorophyll concentration (green line) and water level (red line) during 8 days in winter 2007. (B) Time series of the rate of change in chlorophyll concentration (green line) and the estimated tidal current speed (TC, red line). (C) Wavelet coherence spectrum of the two time series in panel B. Color coding indicates the coherence of the two time series. Arrows indicate the phase angle between fluctuations of the two time series, with arrows pointing to the right representing in-phase fluctuations. See the legend of Fig. 3 for further explanation of wavelet spectra. (D) Relative distribution of phase angles between fluctuations in the rate of change in chlorophyll concentration and fluctuations in tidal current speed. (E) Time series of hourly averaged chlorophyll concentration (green line) and SPM concentration (black line). (F) Wavelet coherence spectrum of the two time series in panel E. (G) Relative distribution of phase angles between fluctuations in chlorophyll concentration and SPM concentration. The phase angle distributions in (D) and (G) are based on the complete time series (2001–2009).

Comparison with the tidal data reveals that chlorophyll concentrations consistently peaked a few hours before high tide and a few hours before low tide (Fig. 5A). The timing suggests that the chlorophyll peaks are generated by enhanced tidal mixing due to high tidal current speeds. To investigate this hypothesis, we calculated the rate at which the water level (W) increased or decreased as a simple proxy of the tidal current speed (TC):
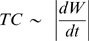



Taking the absolute value (vertical bars) transforms the 12 hour 25 min periodicity of the water level to a 6 hour 12 min periodicity in tidal current speed. Indeed, the rate of change in chlorophyll concentration seems to fluctuate in phase with tidal current speed (Fig. 5B).

This phase relationship can be tested by wavelet coherence analysis, which displays the coherence (colors) and phase angles (arrows) between two fluctuating time series. Bold black lines delineate areas of significant coherence. Arrows pointing to the right indicate in-phase fluctuations. This confirms that the rate of change in chlorophyll concentration fluctuated in phase with tidal current speed at a significant 6-hour periodicity (Fig. 5C). In other words, chlorophyll concentrations increased during high tidal and decreased during low tidal current speeds. Calculation of all significant phase angles in the entire time series of 2001–2009 reveals a dominant phase angle of 310–330° (Fig. 5D), corresponding to a minor phase delay of ∼45 minutes between tidal current speed and the rate of change in chlorophyll concentration. Wavelet coherence analysis also shows significant coherence between the time series of chlorophyll and SPM concentration, which fluctuated in phase at a 6-hour and 12-hour periodicity (Fig. 5E–G).

### Analysis of Daily-averaged Time Series

**Figure 6:**
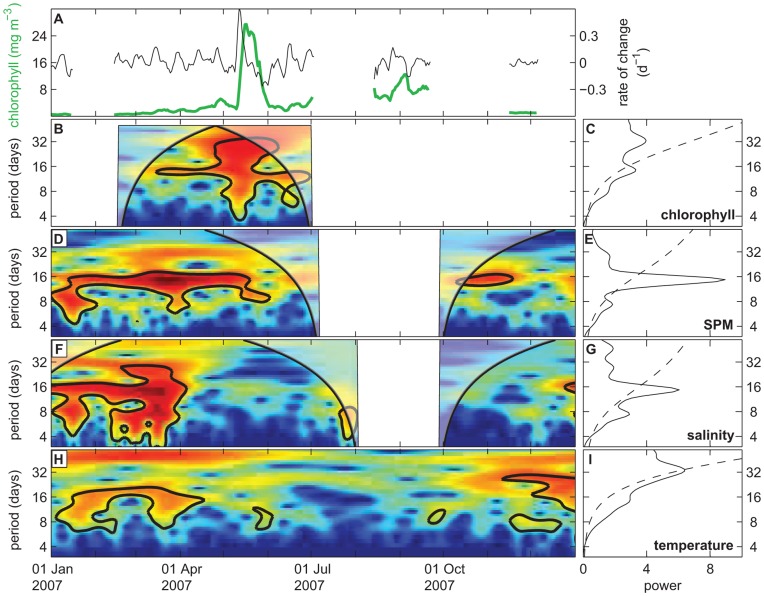
Wavelet analysis of chlorophyll, SPM, salinity and temperature on a daily time scale. (A) Original time series (green line) and transformed time series (rate of change, black line) of daily averaged chlorophyll concentration in the year 2007. (B-I) Wavelet power spectra (left panels) and global wavelet spectra (right panels) of daily averaged data of (B, C) chlorophyll concentration, (D, E) SPM concentration, (F, G) salinity, and (H, I) temperature. The wavelet power spectra show only the year 2007; the global wavelet spectra are based on the complete time series (2001–2009). See the legend of Fig. 3 for an explanation of wavelet power spectra.

In Figure 6, we move away from the within-day variability and focus on longer time scales using the daily-averaged time series of 2007. Comparison of transformed data (black line) and untransformed data (green line) illustrates that expressing the chlorophyll data as relative rates of change suppresses the amplitude of large peaks such as the spring bloom, and uncovers periodic variation in chlorophyll concentration during other times of the year (Fig. 6A). Wavelet analysis reveals significant 15-day periodicity in chlorophyll concentrations during March-April and June (Fig. 6B). In May, the significant area is spread across a wider range of periodicities due to the relatively large amplitude (and, hence, large power) of the spring bloom. Daily-averaged SPM and salinity also show significant 15-day periodicity, at least during part of the year (Figs. 6D,F). Daily-averaged temperature does not show a clear peak at any specific time scale in the wavelet power spectrum (Fig. 6H). Analysis of the entire dataset of 2001–2009, summarized in the global wavelet spectra (Figs. 6C, E, G, I), confirms the 2007 results with significant peaks at a 15-day periodicity for chlorophyll, SPM and salinity.

**Figure 7:**
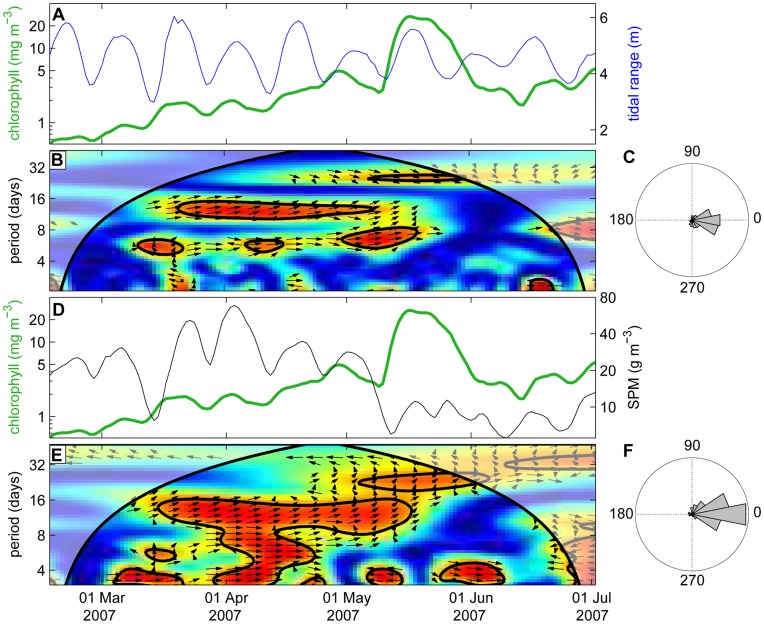
Coherence between fluctuations in chlorophyll, tidal range and SPM on a daily time scale. (A) Time series of daily averaged chlorophyll concentration (green line) and tidal range (blue line) during spring of 2007. (B) Wavelet coherence spectrum of the two time series in panel A. Color coding indicates the coherence of the two time series. Arrows indicate the phase angle between fluctuations of the two time series. See the legend of Fig. 3 for further explanation of wavelet spectra. (C) Relative distribution of phase angles between fluctuations in chlorophyll concentration and tidal range. (D) Time series of daily averaged chlorophyll concentration (green line) and SPM concentration (black line). (E) Wavelet coherence spectrum of the two time series in panel D. (F) Relative distribution of phase angles between fluctuations in chlorophyll concentration and SPM concentration. The phase angle distributions in (C) and (F) are based on the complete time series (2001–2009).

Wavelet coherence analysis shows that the chlorophyll concentration fluctuated in phase with the tidal range (Fig. 7A–C) and SPM concentration (Fig. 7D–F) during most of the year. In 2007, the spring bloom slowly built up over several spring-neap cycles in March and April, and then took off to high chlorophyll concentrations in early May, when the SPM concentration declined much stronger at neap tide than in the preceding months (Fig. 7D; see also Fig. 2A). Interestingly, during this chlorophyll peak in May, the chlorophyll fluctuations lost coherence with the tidal range and SPM fluctuations at a 15-day periodicity (Figs. 7B,E). In a sense, one might say that the spring bloom “escaped” from the spring-neap tidal cycle.

This sequence of events, where a strong decline of the SPM concentration in late April or early May was accompanied by a large spring bloom, was also observed in the years 2001, 2003, 2004 and 2008 (Figs. S2, S3, S4, S5, S6, S7, S8, S9). In all other years the monitoring data contained one or more larger gaps due to fouling of the sensors, such that we could not use those years to reliably assess the full sequence of events during the spring bloom.

## Discussion

### Fluctuations Driven by the Semidiurnal Tidal Cycle

Our results show that short-term fluctuations of coastal phytoplankton were dominated by periodicities of 6 hours 12 min, 12 hours 25 min, and to a lesser extent 24 hours, reflecting the typical periodicities in tidal current speed, horizontal displacement of tidal waters, and the day-night cycle, respectively. Likewise, SPM also fluctuated at 6-hour and 12-hour periodicities, in phase with the chlorophyll fluctuations. One possible explanation for the in-phase fluctuations of chlorophyll and SPM could be that SPM is largely composed of phytoplankton. We therefore estimated the relative contribution of phytoplankton to SPM. Assuming a phytoplankton biomass to carbon ratio of 2.5 [37] and a carbon to chlorophyll ratio of 40 (for marine diatoms in nutrient-rich temperate waters; [38]), then 1 mg m^−3^ of chlorophyll should contribute 100 mg m^−3^ of SPM. In view of the measured concentrations of chlorophyll and SPM in our time series, this implies that phytoplankton comprised less than 5% of the SPM concentration throughout the entire year, except during the spring bloom when phytoplankton increased to ∼20% of total SPM. However, during the spring bloom the phytoplankton dynamics diverged from the SPM dynamics, as the decline in SPM concentration was accompanied by a rise in chlorophyll concentration. For these reasons, the in-phase fluctuations of chlorophyll and SPM cannot be explained by the assumption that SPM consisted mostly of phytoplankton particles.

Instead, we hypothesize that the 6-hour periodicity and in-phase dynamics of chlorophyll and SPM are explained by alternating sinking and vertical mixing of phytoplankton and SPM driven by the tidal cycle. Phytoplankton and SPM both have a particulate nature. Hence, surface concentrations of phytoplankton and SPM will decrease simultaneously due to sedimentation during weak turbulent mixing (i.e., at each high and each low tide slack). Conversely, phytoplankton and SPM will increase simultaneously by resuspension during periods of intense tidal mixing generated by the high current speeds between each high and low tide. This explanation is supported by the in-phase fluctuations of the chlorophyll dynamics and tidal current speed (Fig. 5). In fact, we found a minor phase delay of 45 min between fluctuations of tidal current speed and of chlorophyll concentration, which might reflect the time it takes for high tidal current speeds to mix sedimented phytoplankton cells upwards to the water surface. Resuspension of benthic diatoms by enhanced vertical mixing has also been observed by many other studies, and is often held responsible for much of the chlorophyll variability in shallow estuaries and intertidal areas [23], [39], [40]. Indeed, several of the diatom species dominant at our mooring site can be classified as tychoplankton (e.g. *Cylindrotheca*, *Paralia*, *Plagiogrammopsis*), which combine a benthic and pelagic life style as they commonly settle on the sediment during weak mixing but are resuspended during intense tidal mixing [41]–[43].

Sinking rates of single diatom cells range from near zero to 10 m d^−1^ [44], [45]. Models of coastal phytoplankton using such sinking rates do not predict significant 6-hour periodicity in surface chlorophyll concentrations [46], [47]. This suggests that higher sinking rates are required to explain the observed 6-hour variation in surface chlorophyll concentration. Sinking rates of diatom aggregates are often much higher than for single cells, ranging from 50 to 150 m d^−1^ [48]–[50]. Many of the dominant diatom species observed at station Warp are known to form aggregates with other diatoms and sediment particles, through the production of extracellular polymeric substances and the entanglement of diatom spines [51]–[53]. Aggregate formation is therefore likely to play a role in the 6-hour periodicity of the chlorophyll concentration.

Salinity, nitrate and temperature are not subject to sedimentation and resuspension by tidal mixing. Indeed, salinity, nitrate and temperature showed only minor 6-hour periodicity, but fluctuated predominantly at a periodicity of 12 hours 25 min. This periodicity matches the typical time scale of horizontal displacement of water masses moving back and forth by tidal motion. Horizontal transport of different water masses is further confirmed by the observation that salinity fluctuated in phase with water level, while nitrate fluctuated in anti-phase with water level at a 12-hour 25-min periodicity (Fig. S11). This reflects an enhanced influence of nutrient-rich freshwater from the river Thames at low tide and of less nutrient-rich and more saline North Sea water at high tide. The 12-hour 25-min periodicity was visible in the chlorophyll and SPM data as well, indicating that the chlorophyll and SPM fluctuations were also partly driven by horizontal transport of different water masses.

An additional process contributing to the 12 hour 25 min periodicity might be the vertical excursions of the SmartBuoy, which moved up and down with the water level. This could be particularly relevant in waters with strong vertical gradients of the chlorophyll and SPM concentrations in the upper meters of the water column, where the measurements are made. At station Warp, however, the upper meters of the water column are well mixed (Fig. S1). Therefore, the contribution of the vertical excursions of the instruments to the 12 hour 25 min periodicity seems small in comparison to the horizontal transport processes.

In summer, we also found a 24-hour periodicity in chlorophyll fluorescence, but not in SPM. The 24-hour periodicity was less pronounced than the periodicities at 6 hours 12 min and 12 hours 25 min. Wavelet analysis indicates that this 24-hour periodicity is independent of the tidal periodicity, because the peak of the global power spectrum was located at 24 hours sharp rather than at 24 hours 50 min. This day-night cycle is probably caused by non-photochemical quenching of the fluorescence signal during sunny days. Alternatively, this day-night cycle might reflect diurnal variation in convective mixing driven by thermal micro-stratification during the daytime and surface cooling at night. Van Haren et al. [54] reported similar diurnal variation in chlorophyll fluorescence at the Oystergrounds, in the central North Sea. In addition to chlorophyll fluorescence, they also measured chlorophyll by HPLC, and found that decreasing chlorophyll concentrations at 11 m depth matched the increasing chlorophyll concentrations at 23 m depth during the daytime. Hence, van Haren et al. [54] conclude that diurnal convective mixing led to net sedimentation of phytoplankton at daytime and net resuspension at night. However, contrary to the Oystergrounds, station Warp does not show significant 24-hour periodicity of surface temperatures (Fig. 4H), so we expect that the effect of thermal microstratification is relatively small at station Warp.

### Fluctuations Driven by the Spring-neap Tidal Cycle

Superimposed upon the 6-, 12- and 24-hour periodicities the chlorophyll concentration also fluctuated at a significant 15-day periodicity, in phase with the spring-neap tidal cycle. A possible explanation for the 15-day periodicity in phytoplankton abundance is that the spring-neap cycle modulated the nutrient supply. This mechanism was described by Sharples et al. [20], [55], who found that the upward nitrate flux into the surface layer was much higher during spring tide than during neap tide, and fueled new primary production. However, their measurements were made in a stratified water column of 200 m depth at the shelf edge of the Celtic Sea, where upward mixing of nutrients across the thermocline was driven by an internal tide. In contrast, coastal waters of the southern North Sea are shallow (15 m deep at station Warp) and without thermohaline stratification (Fig. S1). In fact, the nitrate concentration at station Warp was highest during neap tide (Fig. S12), when nutrient-rich freshwater from the river Thames was less intensely mixed with the more saline North Sea water. Because nutrient concentrations fluctuated in anti-phase with the tidal cycle, while chlorophyll fluctuated in phase, it seems unlikely that variation in nutrient availability was the main driver for the phytoplankton fluctuations at the time scale of the spring-neap cycle.

Instead, the in-phase fluctuations of both chlorophyll and SPM with the tidal range suggest that a large fraction of the phytoplankton and SPM sinks to the sediment or deeper water layers during calm conditions at neap tide, while they are resuspended by strong tidal mixing during spring tide. Station Warp is part of a large shallow outer estuary of more than 100 km^2^. Hence, the chlorophyll and SPM fluctuations observed at the time scale of the spring-neap cycle will probably not represent small-scale processes restricted to the local neighborhood of station Warp, but may integrate sedimentation and resuspension over a large area. The 15-day periodicity of the SPM concentration confirms recent spectral analyses of remote sensing data of the North Sea, which indicated spring-neap variations in SPM in the East Anglia plume and Rhine plume [56]. Our interpretation is further supported by McCandliss et al. [24], who investigated the dynamics of suspended particles in coastal waters of the southern North Sea. They observed enhanced particle settling and the formation of a phyto-detrital fluff layer at the sediment surface during neap tide and calm wind conditions. This benthic fluff layer was (partly) resuspended by more turbulent conditions during strong spring tide and storms. Hence, we conclude that the 15-day periodicity in chlorophyll concentration is driven by a similar process of alternating sinking and resuspension as the 6-hour periodicity, integrated over a larger area.

### Seasonal Variation and the Phytoplankton Spring Bloom

Earlier analysis indicated that the spring bloom at our coastal mooring site is triggered by improved light conditions due to a combination of enhanced solar radiation and reduced SPM concentrations in spring [26]. Similar observations have been made in other coastal waters [46], [57], [58]. Our results support these findings, but provide a more detailed picture. The chlorophyll concentration did not increase abruptly in spring, but gradually built up over the course of several spring-neap cycles, with periodic ups and downs coinciding with the ups and downs in SPM concentration (Fig. 7D–F; Figs. S2, S3, S4, S5, S6, S7, S8, S9). At some point in mid spring, SPM concentrations decreased more strongly during neap tide than before and stayed low during the subsequent spring tide, thus creating more favorable light conditions for phytoplankton growth. At this point, the chlorophyll concentration continued to rise and temporarily “escaped” coherence with the spring-neap tidal cycle and SPM fluctuations, and a spring bloom developed.

What caused the conspicuous decline of the SPM concentration in mid spring? It might be induced by a strong reduction in tidal range, weakening tidal mixing and thereby enhancing sedimentation of SPM. For instance, the strong SPM decline in early May 2007 occurred when spring tide coincided with apogee of the moon, resulting in a weak spring tide (Fig. 7). Likewise, the strong SPM decline in the third week of May 2004 and third week of April 2008 also occurred when spring tide coincided with apogee (Figs. S5 and S8). However, in the other years covered by our data set the SPM decline was not associated with weak spring tides (Figs. S2, S3, S4, S5, S6, S7, S8, S9). Strong SPM declines might also be induced by stable weather conditions in spring, with high temperatures and low wind speeds, as in April 2003. Moreover, from the time series it is hard to judge whether chlorophyll concentrations increased because of decreasing SPM concentrations, or vice versa. One may hypothesize that SPM concentrations decreased due to aggregate formation induced by extracellular polymeric substances (EPS) produced by phytoplankton. EPS production by diatoms is known to stabilize intertidal sediments [59], [60]. Aggregate formation, and subsequent sedimentation, has been described as an important mechanism affecting SPM dynamics in estuaries [61] and coastal waters [62]. Hence, possibly, rising phytoplankton concentrations and decreasing SPM concentrations are interacting as a positive feedback loop, accelerating the development of the spring bloom.

The phytoplankton spring bloom resulted in rapid depletion of inorganic nutrients, which was followed by lower phytoplankton concentrations during the remainder of the summer. This classic pattern was also observed in the earlier study of Weston et al. [26] at the same site. Interestingly, after each spring bloom, coherence between the chlorophyll and SPM fluctuations was restored. This indicates that suitable light conditions associated with low SPM concentrations were not able to trigger major phytoplankton blooms during summer, presumably because nutrient limitation or grazing control kept the phytoplankton population in check.

### Implications for Phytoplankton Community Structure

Temporal variation in turbulent mixing may result in temporal variation in the phytoplankton community. For instance, Huisman et al. [63] manipulated the turbulence structure of an entire lake using artificial mixing. This lake experiment changed the phytoplankton community from surface blooms by buoyant cyanobacteria at weak turbulent mixing to dominance by sinking freshwater diatoms and green algae at intense turbulent mixing. Likewise, Lauria et al. [64] observed contrasting species responses to variation in turbulent mixing in a tidal estuary. They found that motile dinoflagellates (*Prorocentrum micans* and *Peridinium trochoideum*) aggregated near the water surface during slack water, while they became homogeneously distributed when tidal mixing intensified. Conversely, large pelagic diatoms (*Coscinodiscus* spp.) relied on enhanced turbulent mixing during ebb and flood currents to prevent sinking from the photic zone. In view of these studies, it seems quite plausible that the 6-hour periodicity in chlorophyll concentration observed in our tidal system may also be accompanied by a concomitant 6-hour periodicity in phytoplankton species composition, due to species-specific differences in vertical dispersal by the tidal cycle.

According to the intermediate disturbance hypothesis, biodiversity will be highest in ecosystems exposed to intermediate frequencies of environmental forcing [65], [66]. At one extreme, rapid environmental fluctuations with a periodicity of 6 hours will be faster than the generation times of most phytoplankton species, and will therefore mainly result in a vertical redistribution of existing populations. At the other extreme, environments that remain constant for several weeks to months provide sufficient time for strong phytoplankton species to displace weaker competitors, thus reducing biodiversity [13], [67], [68]. Fluctuations at intermediate frequencies are sufficiently slow to modify the species composition, yet fast enough to prevent competitive exclusion. This is confirmed by experimental tests of the intermediate disturbance hypothesis, which found highest phytoplankton biodiversity at intermediate disturbance intervals of 1–2 weeks [69], [70]. This time interval is fairly close to the 15-day periodicity of the spring-neap tidal cycle that caused such conspicuous fluctuations in chlorophyll concentration. Hence, we hypothesize that environmental forcing by the spring-neap tidal cycle may favor non-equilibrium coexistence of species that respond differently to tidal mixing, with positive effects on phytoplankton biodiversity. This is supported by observations of non-equilibrium coexistence of diatoms and dinoflagellates in the Gulf of Maine, where diatoms dominated during major spring tides while the dominance shifted to dinoflagellates during neap tides and minor spring tides [71]. High-resolution time series of the phytoplankton species composition, capturing changes in species abundances at 6-hour and 15-day periodicities, will be required to investigate these hypotheses in further detail.

### Implications for Marine Monitoring

The superposition of several periodic fluctuations in chlorophyll concentration and phytoplankton species composition are a challenge for the design of marine monitoring strategies. Measurements taken at fixed time intervals that are not commensurate with the natural frequencies of the tidal cycle may introduce structural biases in monitoring data. For instance, the semidiurnal tidal cycle takes slightly longer than 12 hours. Hence, if measurements are taken, say, once every day at noon, this may erroneously suggest large changes in phytoplankton concentration over a period of several days, while these data simply represent different phases of the semidiurnal tidal cycle. Likewise, the spring-neap cycle takes slightly longer than 14 days. Hence, if a water body is sampled once every two weeks, then the data may erroneously suggest large changes in phytoplankton concentration and phytoplankton species composition over a period of several months, while these data actually reflect different phases of the spring-neap cycle. To avoid such structural biases, the temporal resolution of the measurements should be sufficiently refined to capture the periodicities in the system. In practice, this implies that chlorophyll concentrations should be measured at minimum time intervals of ∼1 hour to capture the semidiurnal tidal cycle. Alternatively, if resources are limited, one might consider measuring less frequently but always at the same moments with respect to both the semidiurnal and spring-neap tidal cycle. In those marine ecosystems where the tidal cycle is a key determinant of chlorophyll variability, it will be difficult to make sense of phytoplankton data obtained from low-frequency sampling programs ignoring the tidal periodicities.

### Conclusions

Our results show that careful investigation of the time scales of population fluctuations in relation to environmental forcing can reveal much information on the underlying processes. We found that phytoplankton fluctuations in the southern North Sea reflect different oscillatory modes of the tidal cycle, including variation in tidal current speeds (6 hours 12 min), horizontal water motion (12 hours 25 min) and the spring-neap tidal cycle (15 days). A weaker 24-hour periodicity was also observed. During most of the year, chlorophyll and SPM concentrations fluctuated in phase with the tidal cyle, indicative of alternating periods of sedimentation and resuspension. However, phytoplankton escaped from the spring-neap tidal cycle in spring, when a strong decline in SPM concentration led to improved light conditions. Hence, fluctuations of coastal phytoplankton were strongly driven by external forcing, but intrinsic population processes took over when growth rates were high during the spring bloom. Our findings illustrate that high-resolution monitoring is required to capture this natural variability, which is considered an essential first step for the reliable detection and prediction of the long-term response of coastal phytoplankton to changing environmental conditions.

## Supporting Information

Figure S1 **Contour plots of water density.** The contour plots are based on CTD profiles taken during service visits to the Smartbuoy from August 2004 to July 2006.
(TIF)

Figure S2 **Time series measured in 2001.** (A) Water level (red line) and tidal range (blue solid line) at station Sheerness. When tidal data at station Sheerness were missing, we show the tidal range at station K13A (blue dashed line) rescaled to match the tidal range at Sheerness. (B) Chlorophyll concentration (green) and SPM concentration (black). (C) Nitrate concentration (dark purple) and light intensity at 1 m depth (pink). (D) Salinity (blue) and water temperature (red). In (B-D), dots show the hourly averages and lines the daily averages.
(TIF)

Figure S3 **Time series measured in 2002.** (A) Water level (red line) and tidal range (blue solid line) at station Sheerness. When tidal data at station Sheerness were missing, we show the tidal range at station K13A (blue dashed line) rescaled to match the tidal range at Sheerness. (B) Chlorophyll concentration (green) and SPM concentration (black). (C) Nitrate concentration (dark purple) and light intensity at 1 m depth (pink). (D) Salinity (blue) and water temperature (red). In (B-D), dots show the hourly averages and lines the daily averages.
(TIF)

Figure S4 **Time series measured in 2003.** (A) Water level (red line) and tidal range (blue solid line) at station Sheerness. (B) Chlorophyll concentration (green) and SPM concentration (black). (C) Nitrate concentration (dark purple) and light intensity at 1 m depth (pink). (D) Salinity (blue) and water temperature (red). In (B-D), dots show the hourly averages and lines the daily averages.
(TIF)

Figure S5 **Time series measured in 2004.** (A) Water level (red line) and tidal range (blue solid line) at station Sheerness. (B) Chlorophyll concentration (green) and SPM concentration (black). (C) Nitrate concentration (dark purple) and light intensity at 1 m depth (pink). (D) Salinity (blue) and water temperature (red). In (B-D), dots show the hourly averages and lines the daily averages.
(TIF)

Figure S6 **Time series measured in 2005.** (A) Water level (red line) and tidal range (blue solid line) at station Sheerness. (B) Chlorophyll concentration (green) and SPM concentration (black). (C) Nitrate concentration (dark purple) and light intensity at 1 m depth (pink). (D) Salinity (blue) and water temperature (red). In (B-D), dots show the hourly averages and lines the daily averages.
(TIF)

Figure S7 **Time series measured in 2006.** (A) Water level (red line) and tidal range (blue solid line) at station Sheerness. When tidal data at station Sheerness were missing, we show the tidal range at station K13A (blue dashed line) rescaled to match the tidal range at Sheerness. (B) Chlorophyll concentration (green) and SPM concentration (black). (C) Nitrate concentration (dark purple) and light intensity at 1 m depth (pink). (D) Salinity (blue) and water temperature (red). In (B-D), dots show the hourly averages and lines the daily averages.
(TIF)

Figure S8 **Time series measured in 2008.** (A) Water level (red line) and tidal range (blue solid line) at station Sheerness. When tidal data at station Sheerness were missing, we show the tidal range at station K13A (blue dashed line) rescaled to match the tidal range at Sheerness. (B) Chlorophyll concentration (green) and SPM concentration (black). (C) Nitrate concentration (dark purple) and light intensity at 1 m depth (pink). (D) Salinity (blue) and water temperature (red). In (B-D), dots show the hourly averages and lines the daily averages.
(TIF)

Figure S9 **Time series measured in 2009.** (A) Water level (red line) and tidal range (blue solid line) at station Sheerness. When tidal data at station Sheerness were missing, we show the tidal range at station K13A (blue dashed line) rescaled to match the tidal range at Sheerness. (B) Chlorophyll concentration (green) and SPM concentration (black). (C) Nitrate concentration (dark purple) and light intensity at 1 m depth (pink). (D) Salinity (blue) and water temperature (red). In (B-D), dots show the hourly averages and lines the daily averages.
(TIF)

Figure S10 **Coherence between fluctuations in light attenuation and SPM on a daily time scale.** (A) Time series of the light attenuation coefficient Kd (pink line) and SPM concentration (black line) using daily averaged data of spring 2005. Kd is calculated from PAR measurements at 1 m and 2 m depth. (B) Wavelet coherence spectrum of the two time series in panel A. Color coding indicates the coherence of the two time series. Arrows indicate the phase angle between fluctuations of the two time series. See the legend of Fig. 3 for further explanation of wavelet spectra. (C) Relative distribution of phase angles between fluctuations in Kd and SPM concentration, based on the complete time series (2001–2009).
(TIF)

Figure S11 **Coherence between fluctuations in water level, salinity and nitrate on an hourly time scale.** (A) Time series of water level (red line) and salinity (blue line) on an hourly time scale during 8 days in winter 2007. (B) Wavelet coherence spectrum of the two time series in panel A. Color coding indicates the coherence of the two time series. Arrows indicate the phase angle between fluctuations of the two time series. See the legend of Fig. 3 for further explanation of wavelet spectra. (C) Relative distribution of phase angles between fluctuations in water level and salinity. (D) Time series of water level (red line) and nitrate concentration (purple line) on an hourly time scale. (E) Wavelet coherence spectrum of the two time series in panel D. (F) Relative distribution of phase angles between fluctuations in water level and nitrate concentration. The phase angle distributions in (C) and (F) are based on the complete time series (2001–2009).
(TIF)

Figure S12 **Coherence between fluctuations in nitrate concentration, tidal range and salinity on a daily time scale.** (A) Time series of nitrate concentration (thick purple line) and tidal range (thin blue line) on a daily time scale during spring of 2007. (B) Wavelet coherence spectrum of the two time series in panel A. Color coding indicates the coherence of the two time series. Arrows indicate the phase angle between fluctuations of the two time series. See the legend of Fig. 3 for further explanation of wavelet spectra. (C) Relative distribution of phase angles between fluctuations in nitrate concentration and tidal range. (D) Time series of nitrate concentration (thick purple line) and salinity (thin blue line) on a daily time scale. (E) Wavelet coherence spectrum of the two time series in panel D. (F) Relative distribution of phase angles between fluctuations in nitrate concentration and salinity. The phase angle distributions in (C) and (F) are based on the complete time series (2001–2009).
(TIF)
