# Supplementary figures and images for: Dancing with the Tides: Fluctuations of Coastal Phytoplankton Orchestrated by Different Oscillatory Modes of the Tidal Cycle
Source: PLoS One. 2012 Nov 14;7(11):e49319. doi: 10.1371/journal.pone.0049319 (PMC3498149; doi:10.1371/journal.pone.0049319)

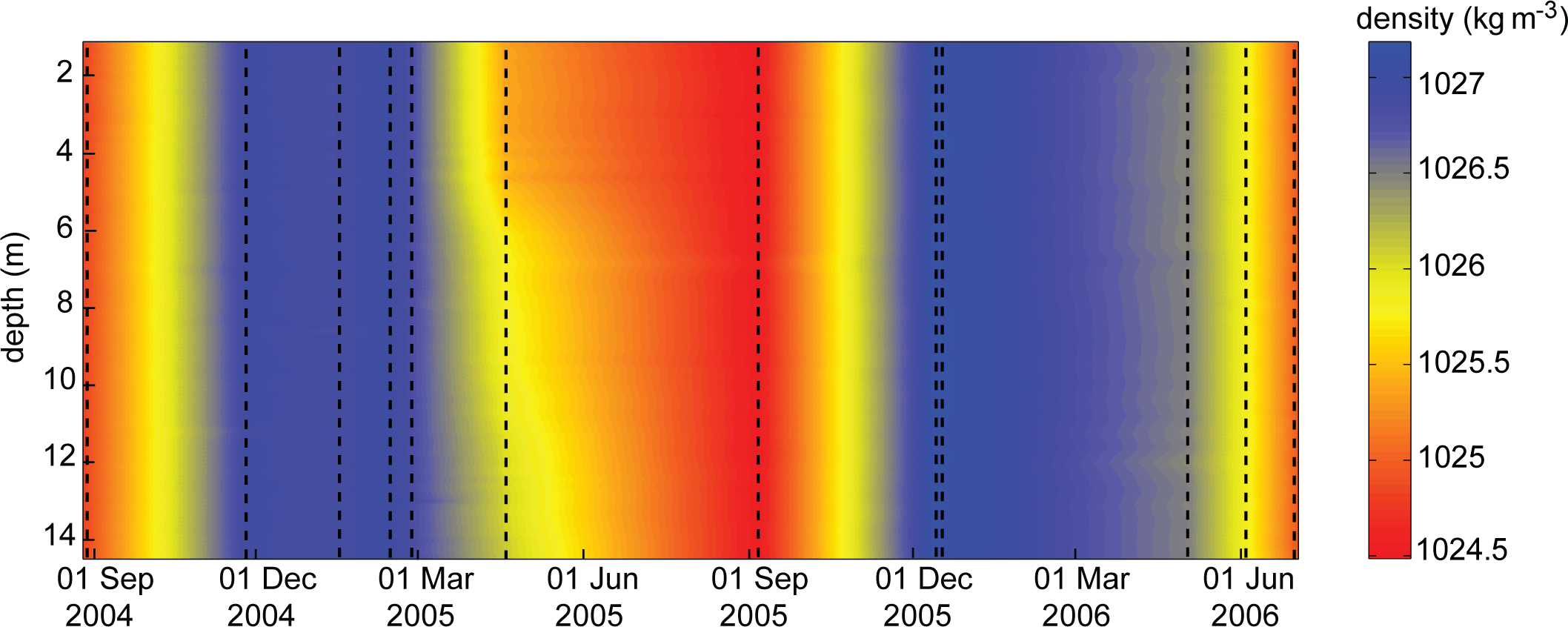

Supplement: Figure S1 — Contour plots of water density. The contour plots are based on CTD profiles taken during service visits to the Smartbuoy from August 2004 to July 2006. (TIF) [file pone.0049319.s001.tif]

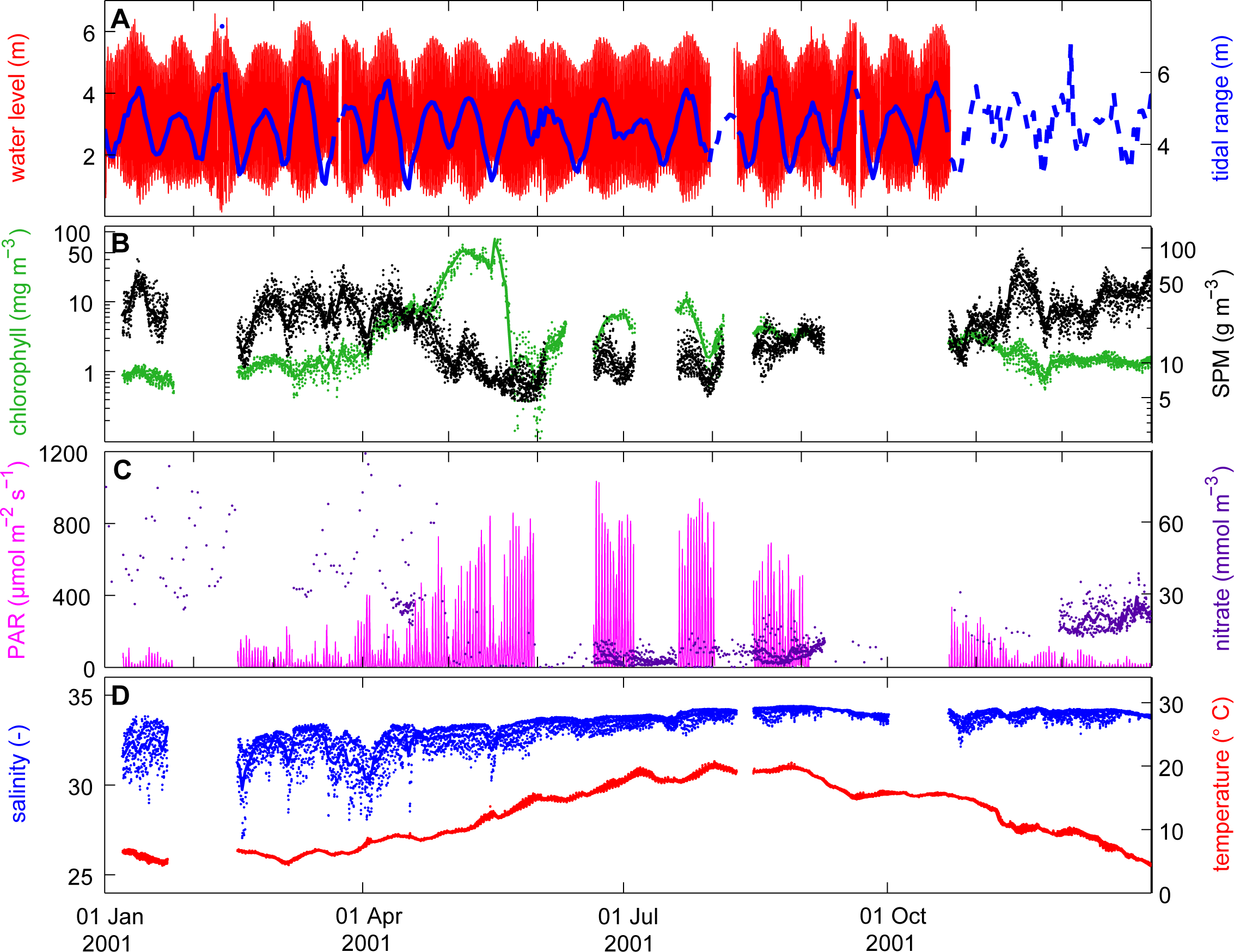

Supplement: Figure S2 — Time series measured in 2001. (A) Water level (red line) and tidal range (blue solid line) at station Sheerness. When tidal data at station Sheerness were missing, we show the tidal range at station K13A (blue dashed line) rescaled to match the tidal range at Sheerness. (B) Chlorophyll concentration (green) and SPM concentration (black). (C) Nitrate concentration (dark purple) and light intensity at 1 m depth (pink). (D) Salinity (blue) and water temperature (red). In (B-D), dots show the hourly averages and lines the daily averages. (TIF) [file pone.0049319.s002.tif]

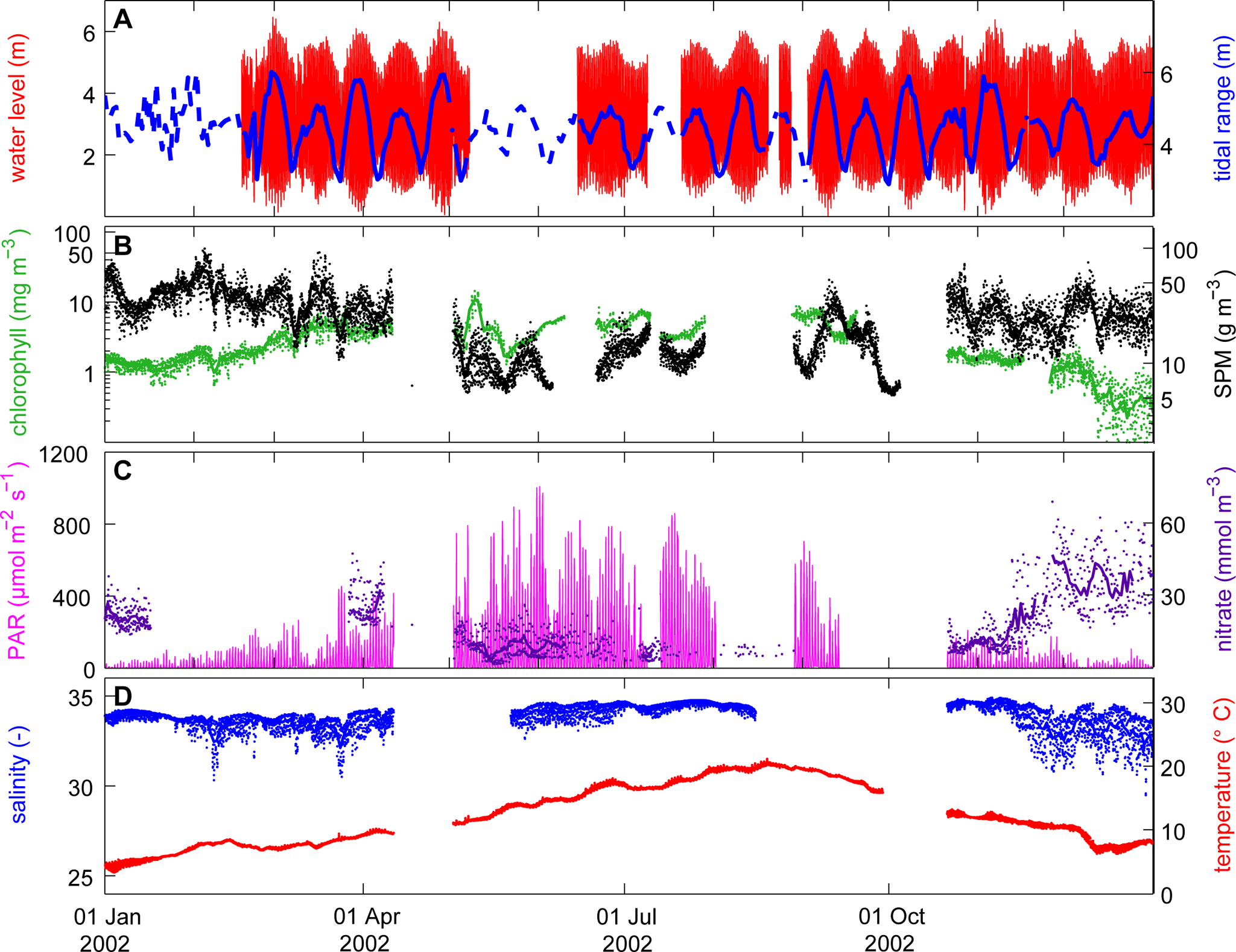

Supplement: Figure S3 — Time series measured in 2002. (A) Water level (red line) and tidal range (blue solid line) at station Sheerness. When tidal data at station Sheerness were missing, we show the tidal range at station K13A (blue dashed line) rescaled to match the tidal range at Sheerness. (B) Chlorophyll concentration (green) and SPM concentration (black). (C) Nitrate concentration (dark purple) and light intensity at 1 m depth (pink). (D) Salinity (blue) and water temperature (red). In (B-D), dots show the hourly averages and lines the daily averages. (TIF) [file pone.0049319.s003.tif]

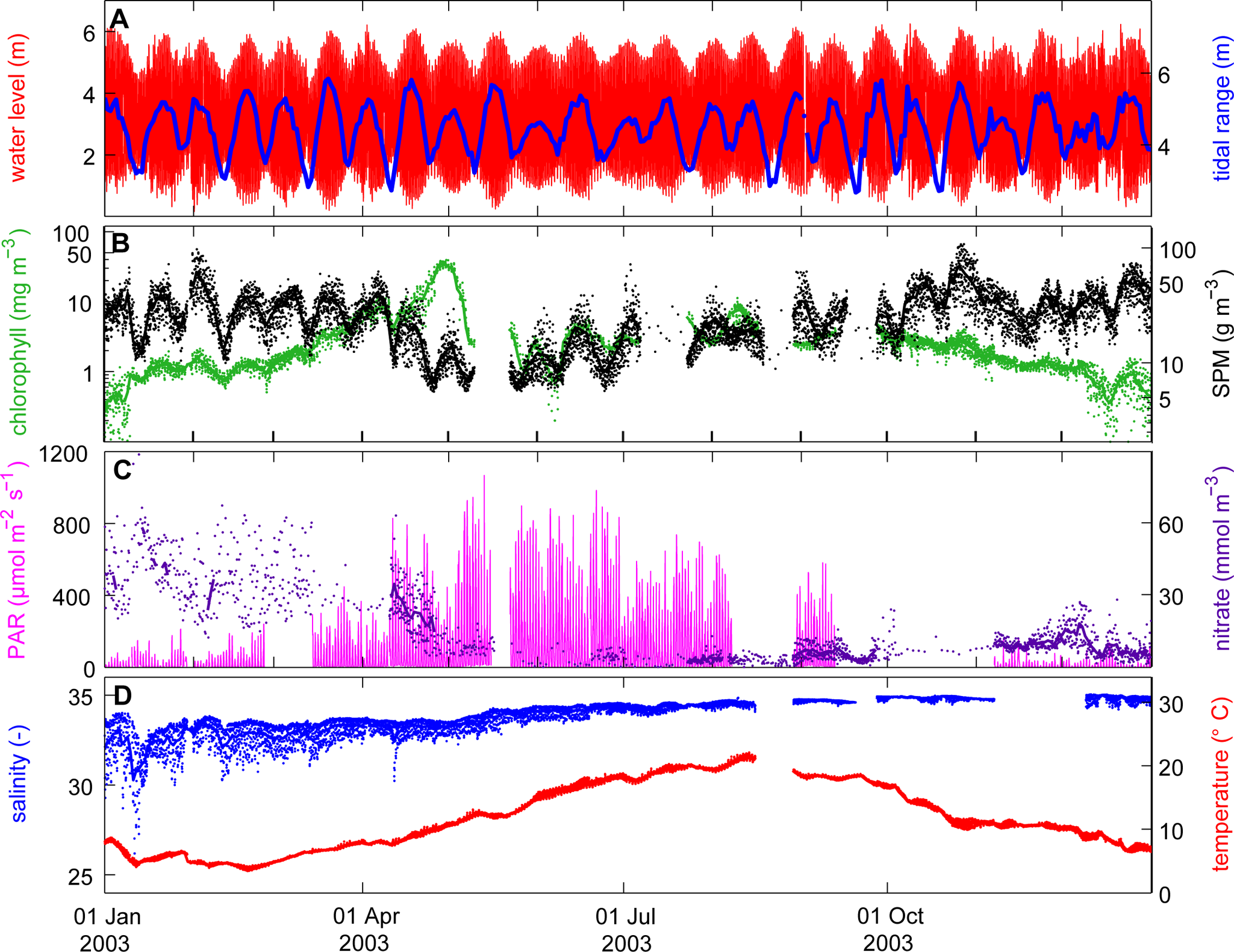

Supplement: Figure S4 — Time series measured in 2003. (A) Water level (red line) and tidal range (blue solid line) at station Sheerness. (B) Chlorophyll concentration (green) and SPM concentration (black). (C) Nitrate concentration (dark purple) and light intensity at 1 m depth (pink). (D) Salinity (blue) and water temperature (red). In (B-D), dots show the hourly averages and lines the daily averages. (TIF) [file pone.0049319.s004.tif]

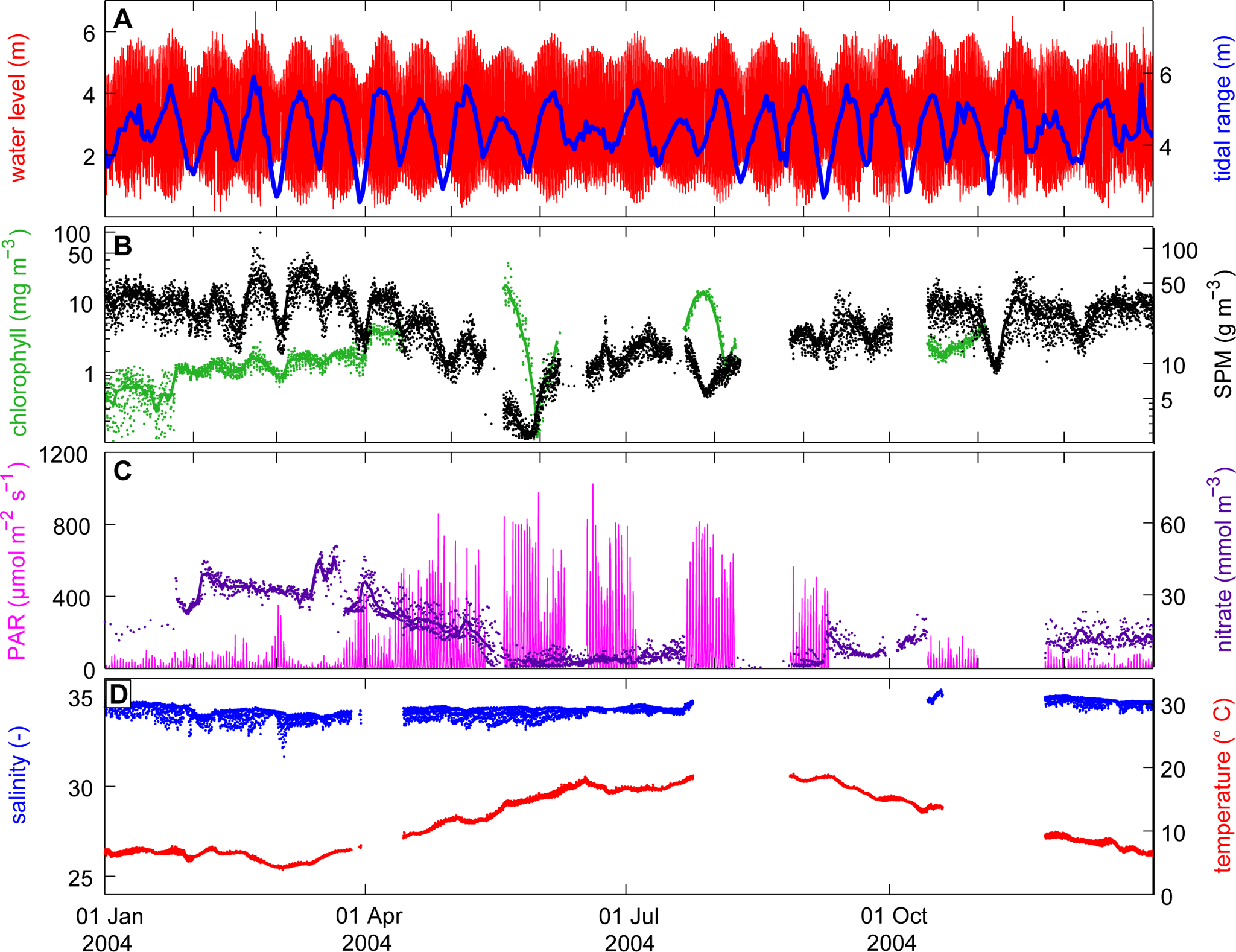

Supplement: Figure S5 — Time series measured in 2004. (A) Water level (red line) and tidal range (blue solid line) at station Sheerness. (B) Chlorophyll concentration (green) and SPM concentration (black). (C) Nitrate concentration (dark purple) and light intensity at 1 m depth (pink). (D) Salinity (blue) and water temperature (red). In (B-D), dots show the hourly averages and lines the daily averages. (TIF) [file pone.0049319.s005.tif]

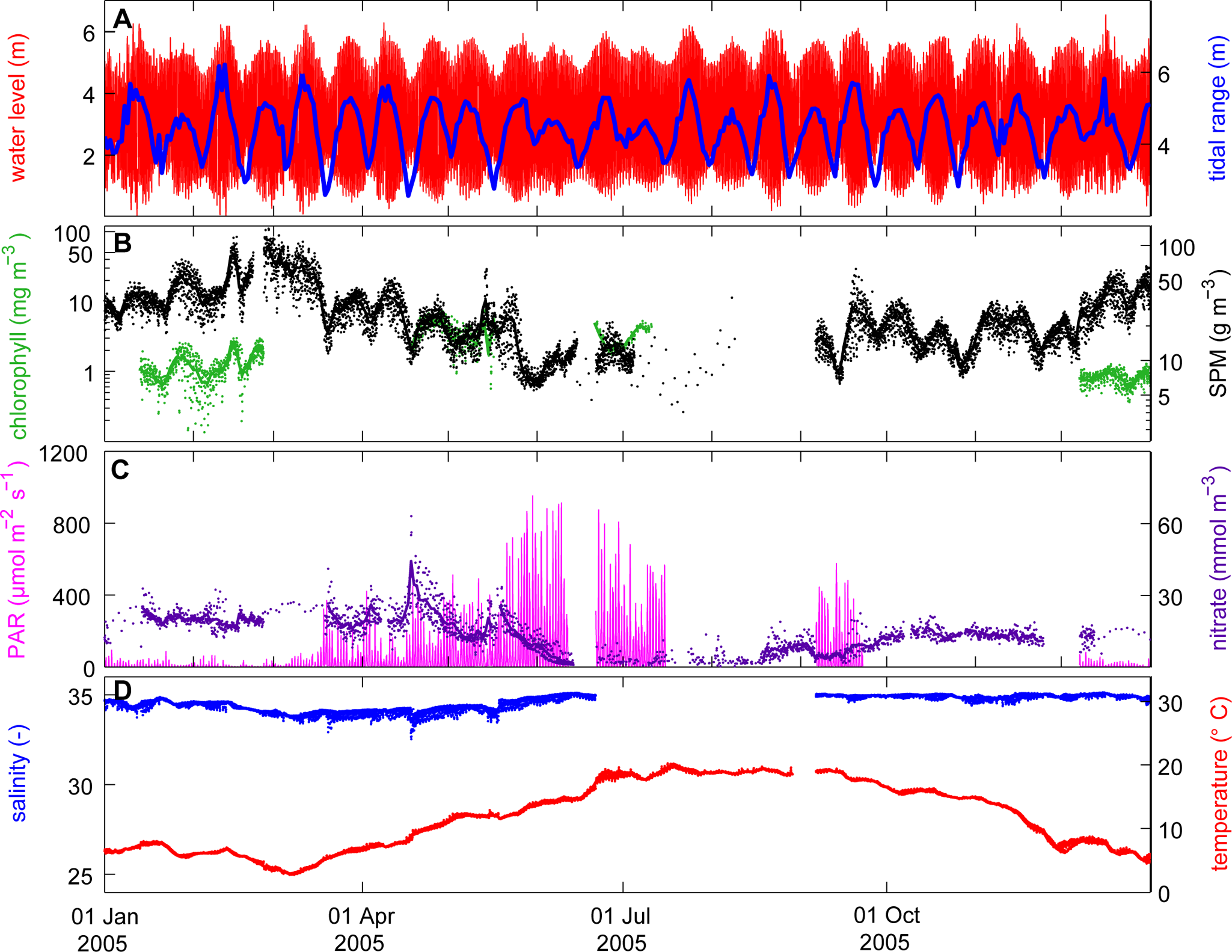

Supplement: Figure S6 — Time series measured in 2005. (A) Water level (red line) and tidal range (blue solid line) at station Sheerness. (B) Chlorophyll concentration (green) and SPM concentration (black). (C) Nitrate concentration (dark purple) and light intensity at 1 m depth (pink). (D) Salinity (blue) and water temperature (red). In (B-D), dots show the hourly averages and lines the daily averages. (TIF) [file pone.0049319.s006.tif]

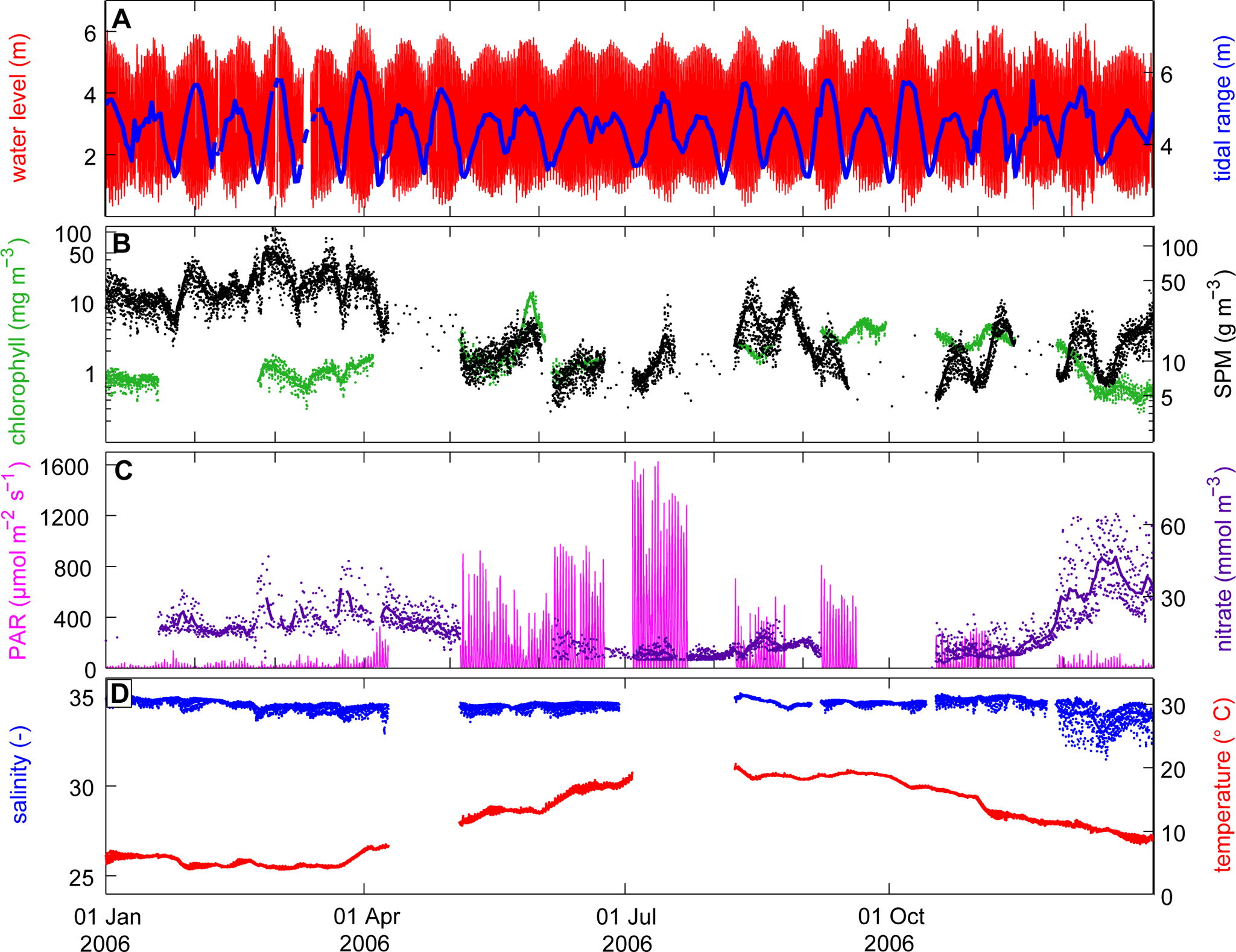

Supplement: Figure S7 — Time series measured in 2006. (A) Water level (red line) and tidal range (blue solid line) at station Sheerness. When tidal data at station Sheerness were missing, we show the tidal range at station K13A (blue dashed line) rescaled to match the tidal range at Sheerness. (B) Chlorophyll concentration (green) and SPM concentration (black). (C) Nitrate concentration (dark purple) and light intensity at 1 m depth (pink). (D) Salinity (blue) and water temperature (red). In (B-D), dots show the hourly averages and lines the daily averages. (TIF) [file pone.0049319.s007.tif]

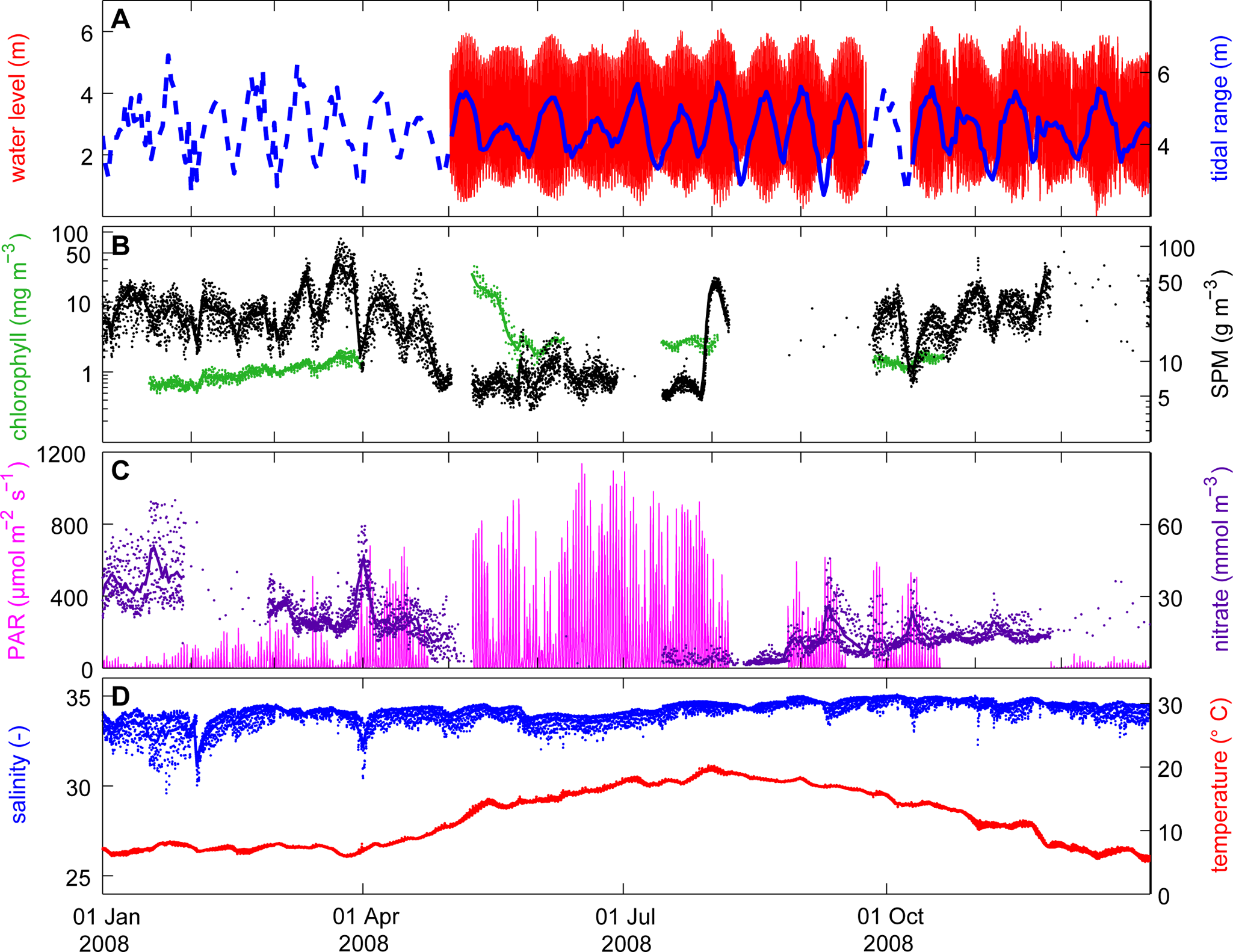

Supplement: Figure S8 — Time series measured in 2008. (A) Water level (red line) and tidal range (blue solid line) at station Sheerness. When tidal data at station Sheerness were missing, we show the tidal range at station K13A (blue dashed line) rescaled to match the tidal range at Sheerness. (B) Chlorophyll concentration (green) and SPM concentration (black). (C) Nitrate concentration (dark purple) and light intensity at 1 m depth (pink). (D) Salinity (blue) and water temperature (red). In (B-D), dots show the hourly averages and lines the daily averages. (TIF) [file pone.0049319.s008.tif]

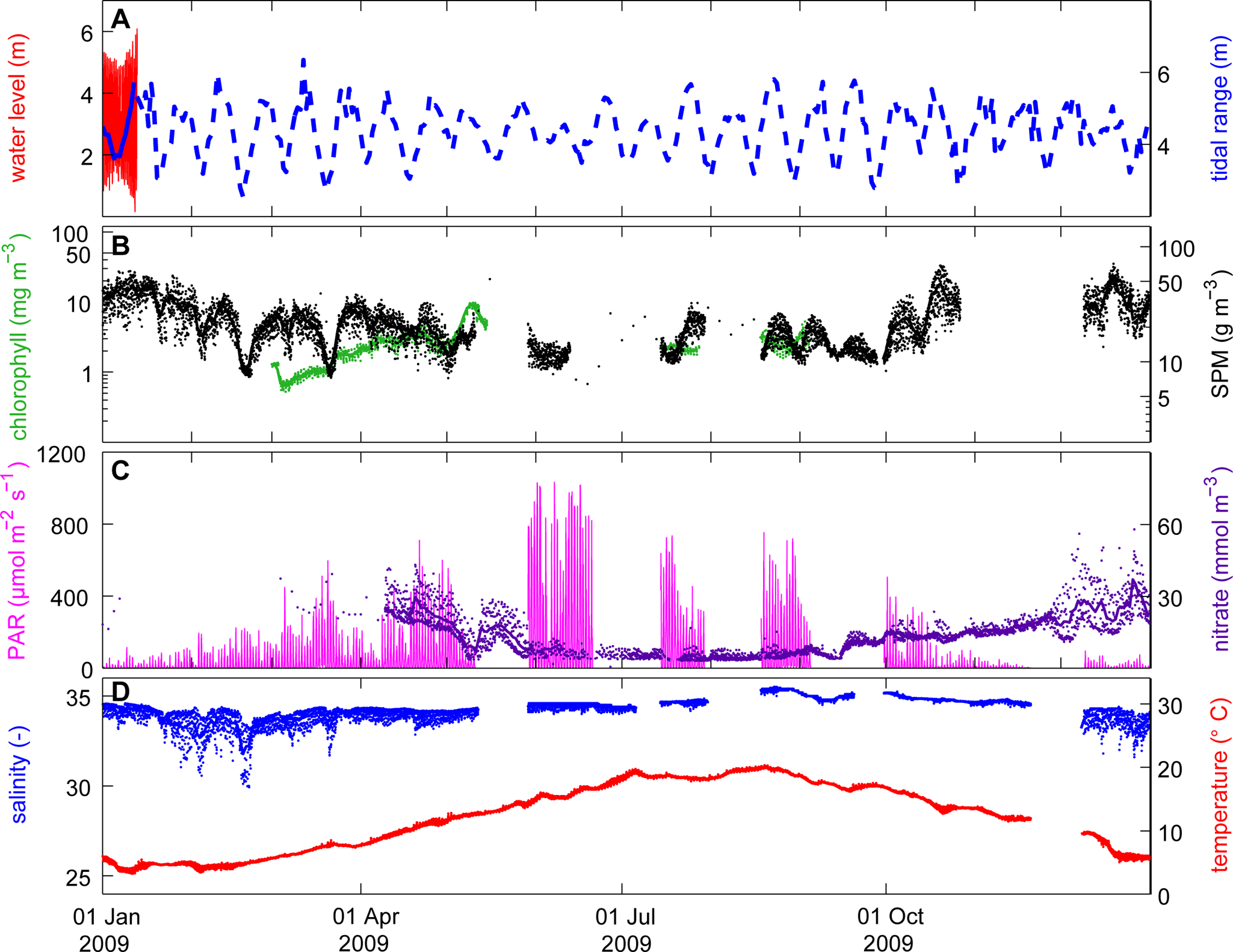

Supplement: Figure S9 — Time series measured in 2009. (A) Water level (red line) and tidal range (blue solid line) at station Sheerness. When tidal data at station Sheerness were missing, we show the tidal range at station K13A (blue dashed line) rescaled to match the tidal range at Sheerness. (B) Chlorophyll concentration (green) and SPM concentration (black). (C) Nitrate concentration (dark purple) and light intensity at 1 m depth (pink). (D) Salinity (blue) and water temperature (red). In (B-D), dots show the hourly averages and lines the daily averages. (TIF) [file pone.0049319.s009.tif]

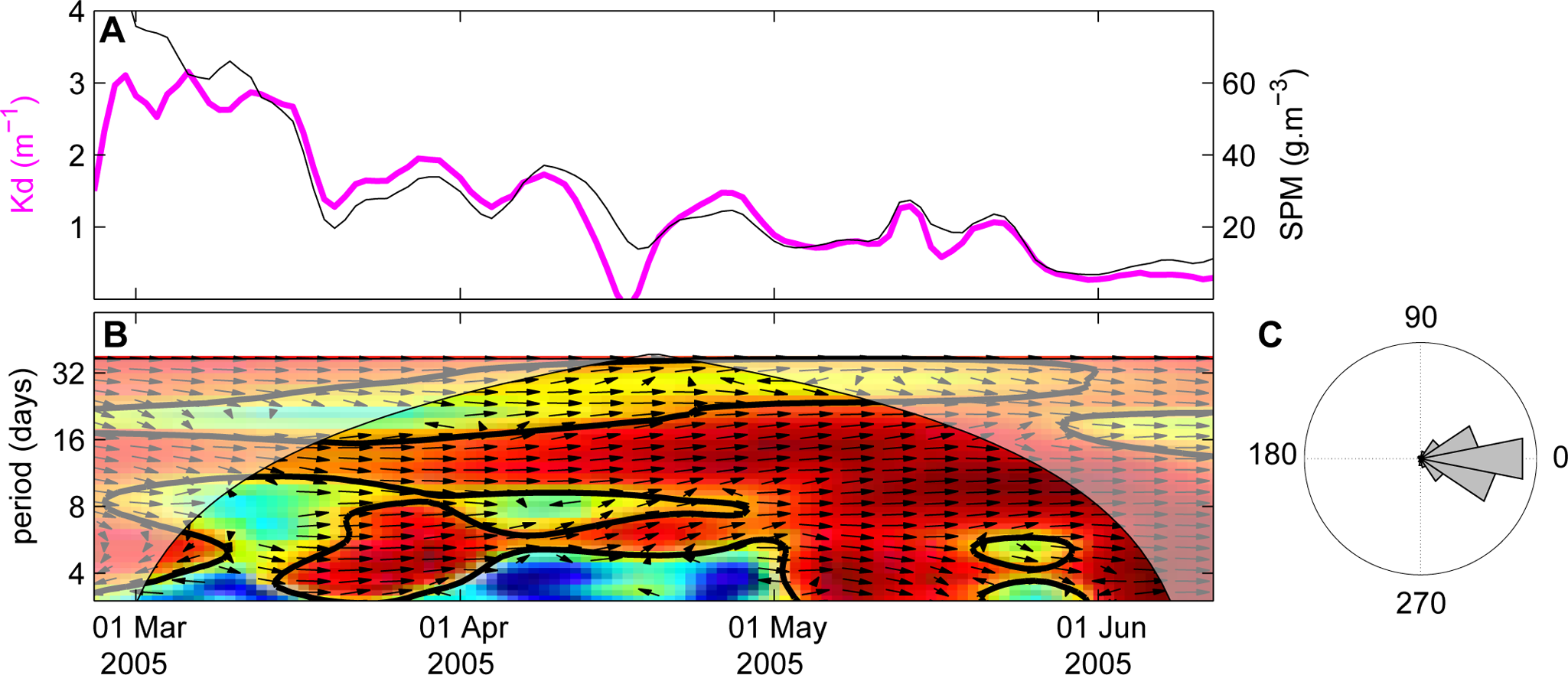

Supplement: Figure S10 — Coherence between fluctuations in light attenuation and SPM on a daily time scale. (A) Time series of the light attenuation coefficient Kd (pink line) and SPM concentration (black line) using daily averaged data of spring 2005. Kd is calculated from PAR measurements at 1 m and 2 m depth. (B) Wavelet coherence spectrum of the two time series in panel A. Color coding indicates the coherence of the two time series. Arrows indicate the phase angle between fluctuations of the two time series. See the legend of Fig. 3 for further explanation of wavelet spectra. (C) Relative distribution of phase angles between fluctuations in Kd and SPM concentration, based on the complete time series (2001–2009). (TIF) [file pone.0049319.s010.tif]

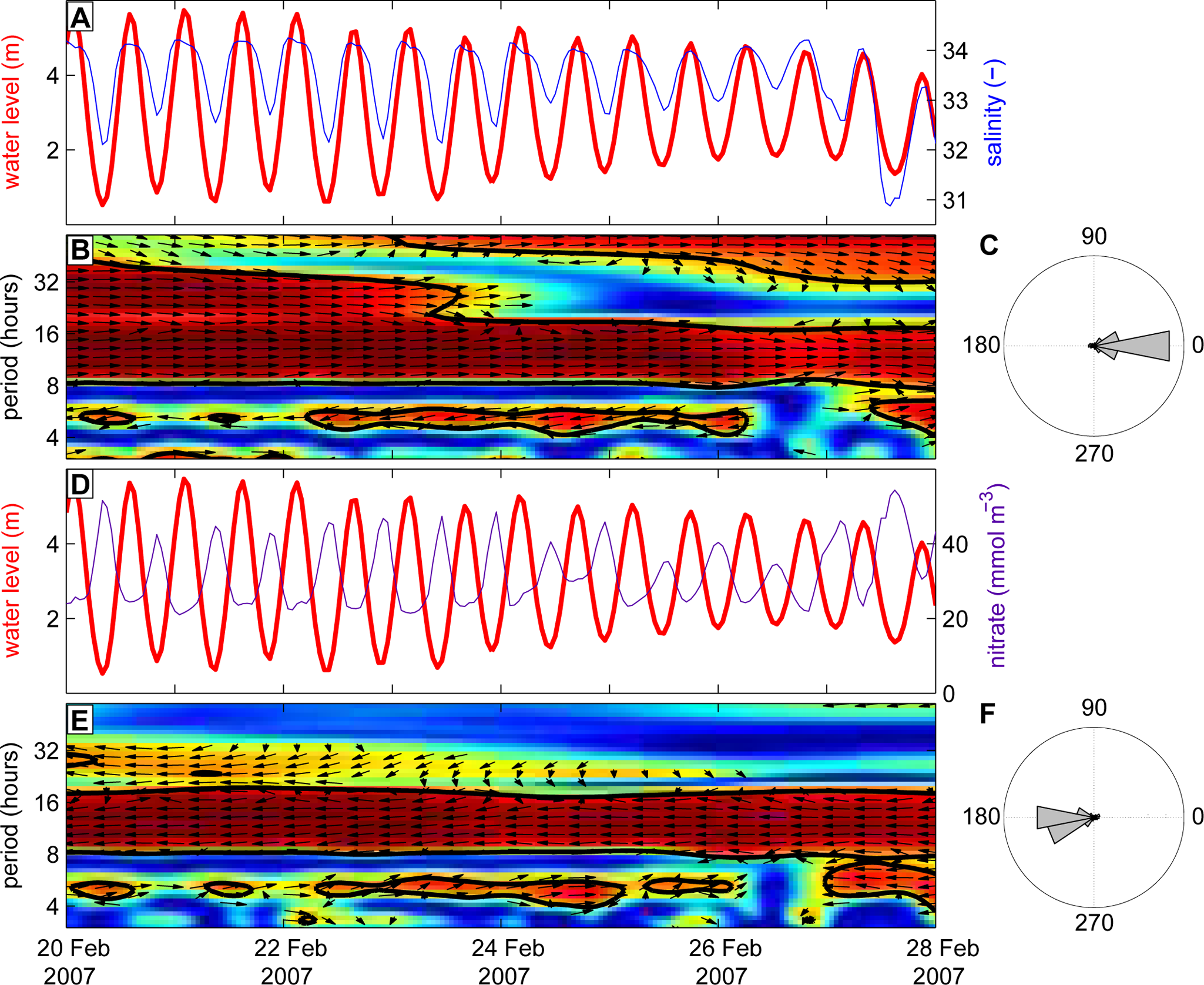

Supplement: Figure S11 — Coherence between fluctuations in water level, salinity and nitrate on an hourly time scale. (A) Time series of water level (red line) and salinity (blue line) on an hourly time scale during 8 days in winter 2007. (B) Wavelet coherence spectrum of the two time series in panel A. Color coding indicates the coherence of the two time series. Arrows indicate the phase angle between fluctuations of the two time series. See the legend of Fig. 3 for further explanation of wavelet spectra. (C) Relative distribution of phase angles between fluctuations in water level and salinity. (D) Time series of water level (red line) and nitrate concentration (purple line) on an hourly time scale. (E) Wavelet coherence spectrum of the two time series in panel D. (F) Relative distribution of phase angles between fluctuations in water level and nitrate concentration. The phase angle distributions in (C) and (F) are based on the complete time series (2001–2009). (TIF) [file pone.0049319.s011.tif]

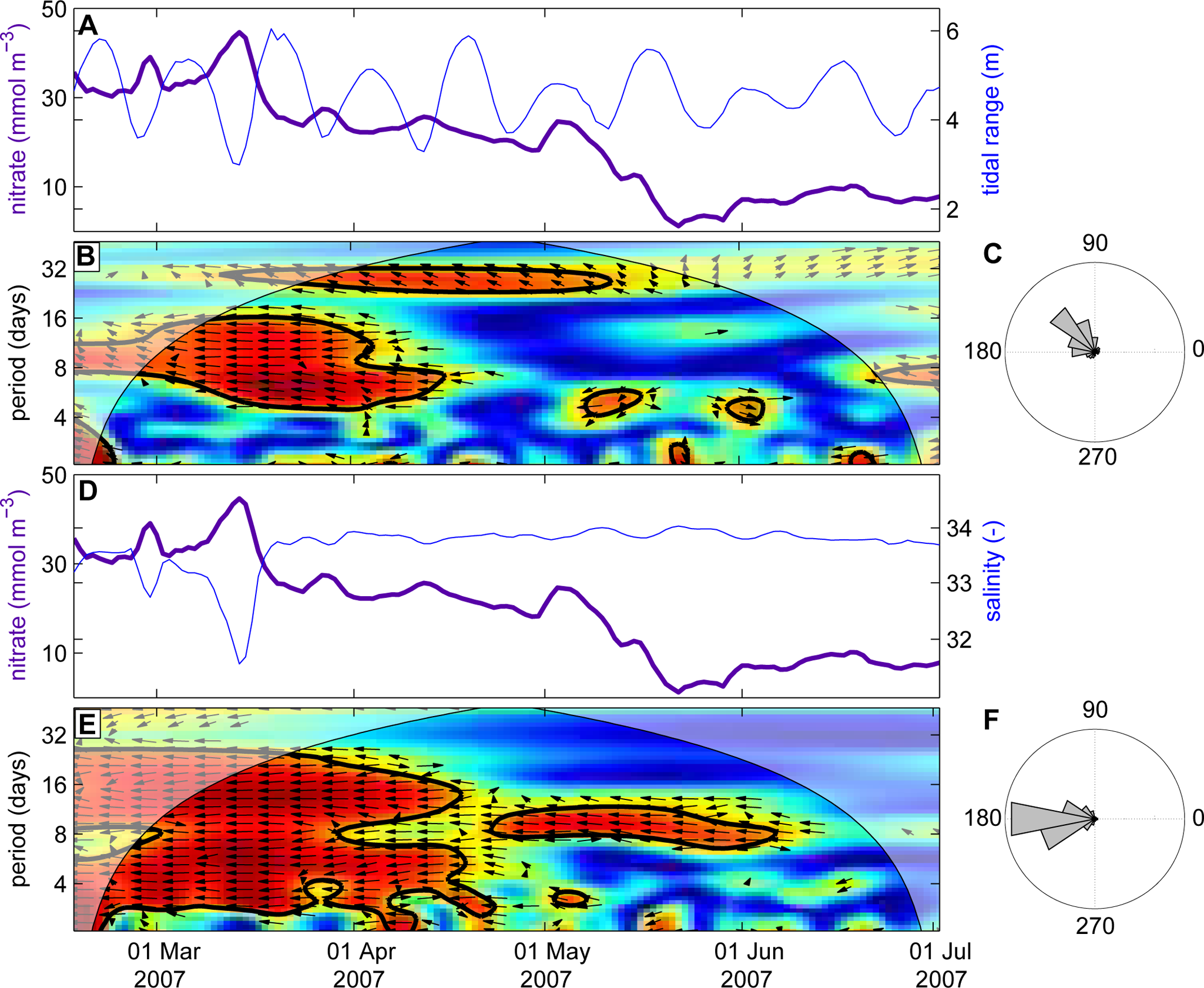

Supplement: Figure S12 — Coherence between fluctuations in nitrate concentration, tidal range and salinity on a daily time scale. (A) Time series of nitrate concentration (thick purple line) and tidal range (thin blue line) on a daily time scale during spring of 2007. (B) Wavelet coherence spectrum of the two time series in panel A. Color coding indicates the coherence of the two time series. Arrows indicate the phase angle between fluctuations of the two time series. See the legend of Fig. 3 for further explanation of wavelet spectra. (C) Relative distribution of phase angles between fluctuations in nitrate concentration and tidal range. (D) Time series of nitrate concentration (thick purple line) and salinity (thin blue line) on a daily time scale. (E) Wavelet coherence spectrum of the two time series in panel D. (F) Relative distribution of phase angles between fluctuations in nitrate concentration and salinity. The phase angle distributions in (C) and (F) are based on the complete time series (2001–2009). (TIF) [file pone.0049319.s012.tif]
